# “Repair Me if You Can”: Membrane Damage, Response, and Control from the Viral Perspective

**DOI:** 10.3390/cells9092042

**Published:** 2020-09-07

**Authors:** Coralie F. Daussy, Harald Wodrich

**Affiliations:** Microbiologie Fondamentale et Pathogénicité, MFP CNRS UMR 5234, University of Bordeaux, 146 rue Leo Saignat, 33076 Bordeaux, France; coralie.daussy@u-bordeaux.fr

**Keywords:** membrane damage, antiviral autophagy, inflammation, galectin, virus entry, interferon, bacterial invasion, adenovirus, lysophagy, ESCRT machinery

## Abstract

Cells are constantly challenged by pathogens (bacteria, virus, and fungi), and protein aggregates or chemicals, which can provoke membrane damage at the plasma membrane or within the endo-lysosomal compartments. Detection of endo-lysosomal rupture depends on a family of sugar-binding lectins, known as galectins, which sense the abnormal exposure of glycans to the cytoplasm upon membrane damage. Galectins in conjunction with other factors orchestrate specific membrane damage responses such as the recruitment of the endosomal sorting complex required for transport (ESCRT) machinery to either repair damaged membranes or the activation of autophagy to remove membrane remnants. If not controlled, membrane damage causes the release of harmful components including protons, reactive oxygen species, or cathepsins that will elicit inflammation. In this review, we provide an overview of current knowledge on membrane damage and cellular responses. In particular, we focus on the endo-lysosomal damage triggered by non-enveloped viruses (such as adenovirus) and discuss viral strategies to control the cellular membrane damage response. Finally, we debate the link between autophagy and inflammation in this context and discuss the possibility that virus induced autophagy upon entry limits inflammation.

## 1. Introduction

Cellular membranes are selective, permeable barriers, consisting of a phospholipid bilayer. They operate by physically separating two compartments within the cell or separate the contents of the cell from the external environment, and they regulate the exchange of chemicals, ions, and biomolecules across them. Cellular membranes thus define organelles and delineate vesicles in intracellular cargo transport systems, such as the endo-lysosomal or the exocytosis pathway. They are both fluid and dynamic, as well as solid and impermeable if necessary [1]. Therefore, membrane integrity is an essential part of cellular homeostasis, which is constantly challenged by pathogens (bacteria, virus, fungi), protein aggregates, or chemicals. Cells have evolutionary conserved surveillance systems to detect and respond to membrane damage and secure cell survival. As outlined in this review, a hallmark of membrane damage is the transient exposure of complex glycosylated proteins in the cytosolic compartment, which are detected by galectins (Gal). Galectins are small cytosolic carbohydrate binding proteins, which cluster at the site of membrane damage. Galectins also coordinate differential responses such as autophagy, to remove membrane remnants [2,3], and the activation of the endosomal sorting complex required for transport (ESCRT) machinery, favoring membrane repair [4,5]. There is increasing evidence that pathogens, including viruses, take control of the cell response to membrane damage and the associated machinery for their own benefit. Therefore, provoking membrane damage upon infection may not be just an accidental by-product of the pathogen entry process. Instead, eliciting membrane damage could be purposeful and part of a sophisticated entry strategy. This review will discuss this possibility and provide an overview of current knowledge on the relationship between membrane damage, cellular response, and pathogen entry. While briefly summarizing observations made for bacterial pathogens and membrane damage in general, our focus is on the entry of the membrane lytic adenovirus (Ad), and other non-enveloped viruses. We discuss the limited existing knowledge about how viruses manipulate and exploit the cellular membrane damage response. We apologize in advance to the many authors who contribute to the emerging field of membrane damage and whose work we cannot cite due to limited space.

### 1.1. Virus Inflicted Membrane Damage

**Figure 1:**
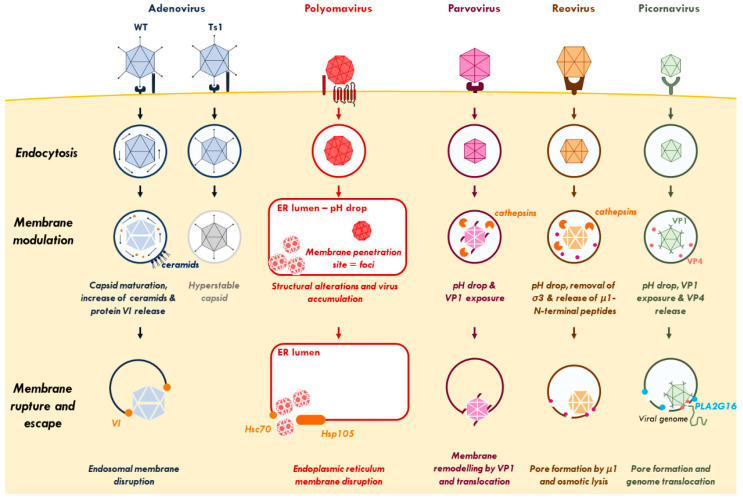
Virus-inflicted membrane damage. After binding to cell-surface receptors, viruses are internalized through endocytosis. Once in the endosome, adenovirus capsid undergoes partial disassembly and releases protein VI. The increase of ceramide concentration enhances the binding of protein VI to the endosomal membrane and its subsequent rupture. Polyomavirus-containing endosomes are targeted to the endoplasmic reticulum (ER) where the virus undergoes conformational changes to penetrate the ER-membrane and escape to the cytosol. Parvovirus and reovirus require a pH drop and the action of endosomal cathepsins to induce conformational rearrangements, disrupt the endosome, and reach the cytosol. After endocytosis and conformational changes, picornaviruses rely on a cellular lipid-modifying enzyme (PLA2G16) to facilitate the translocation of its genome via selective pores across the endosomal membrane. See Section 1 for further details. Abbreviations: ER, endoplasmic reticulum; Hsc70, Heat shock cognate 71 kDa protein; Hsp105, Heat shock protein 105 kDa; PLA2G16, phospholipase A2 group XVI.

Most viruses are taken up by receptor-mediated endocytosis and enter cells through the endo-lysosomal compartment. From here, they have to penetrate into the cytosol to reach their site of replication. For most RNA viruses, replication takes place in the cytosol, while most DNA viruses replicate in the nucleus. The endosome is a dynamic entry compartment, and while it provides some protection from cytosolic innate immune sensors, viruses need to escape before they are either sorted to the lysosome or recycled back to the cell surface [6]. Most enveloped viruses fuse their lipid shell (viral envelope) with the endosomal membrane and release their capsid to the cytosol. This strategy is efficient and topologically simple: it does, *a priori*, not require any kind of membrane rupture [7]. In contrast, non-enveloped viruses, with their hydrophilic capsids, have to cross cell membranes by inflicting membrane damage. This approach risks exposure of the luminal content of the endosome to the cytosol, which can trigger inflammation. To penetrate the cellular membrane, most non-enveloped viruses undergo conformational changes and/or proteolytic processing that allow them to use membrane lytic/modulating factors. Strategies include lipid modification, pore insertion, or large-scale membrane disruption (Figure 1). Still, not much is known about the structural and mechanistic details of the process how non-enveloped viruses inflict membrane damage to cross membranes and even less is known how they engage in the cellular response [8,9].

#### 1.1.1. Adenovirus

Adenoviruses (Ad) are among the best-studied non-enveloped viruses for membrane penetration. Their ~90 nm capsids enter target cells following receptor binding through clathrin-mediated endocytosis. Most Ads rapidly escape the endosomal compartment to avoid lysosomal sorting and use cytosolic motors to reach the nucleus for replication. Adenovirus endosome penetration was shown through co-uptake of non-membrane permeable substrates [10,11]. Observations made with the thermosensitive mutant virus Ad2/5 *ts1* showed that membrane penetration is an essential step in the infection process [12,13,14]. This *ts1* mutant fails to package the adenoviral protease into the capsid. This failure prevents capsid maturation by cleavage of precursor proteins, resulting in hyperstable and non-infectious particles [15,16,17]. Adenovirus capsid maturation is required for the release of the internal membrane lytic capsid protein VI upon virus entry. What triggers protein VI release from the capsid is not known, but it may involve disassembly cues during initial cell binding [14,18,19,20,21]. Protein VI encodes an amphipathic helix with membrane binding and lytic activity [13,22] and mutations in the amphipathic helix strongly reduced both its membrane lytic activity and viral infectivity [23]. Adenovirus inflicted membrane damage creates openings large enough for cytosolic delivery of 70 kDa dextrans or 25 nm parvovirus particles [10,11]. Electron microscopy images of Ad particles in partially disrupted endosomes show large physical openings in the endosome plugged by the virus [3]. High-resolution fluorescence microscopy images suggest localized protein VI release from the capsid at the membrane damage site [24]. Ceramides have been proposed to increase protein VI membrane affinity, showing the importance of the local lipid composition at the membrane penetration site [19]. Protein VI release also plays a role in virus escape from endosomes by counteracting cellular autophagy, which is normally mounted in response to membrane damage as detailed in Section 2 [3].

#### 1.1.2. Polyomavirus

Polyomaviruses (e.g., BK virus and simian virus 40) are small non-enveloped DNA viruses with a diameter of ~45 nm. Particles enter by endocytosis and are transported to the endoplasmic reticulum (ER), from where they reach the cytosol. To penetrate the membrane, particles undergo conformational changes triggered by pH drop and recruited cellular chaperones [25]. Exposure of the N-terminus of the major capsid protein VP1 uncovers a hidden myristylated domain in the internal capsid protein VP2. This structural alteration makes the virus particle significantly more hydrophobic and primes the capsid for membrane binding and particle translocation [26]. Penetration sites are located at the ER membrane and form distinct, virus-induced foci. Several cellular proteins, including chaperones Heat shock cognate 71 kDa protein (Hsc70) and human heat shock protein 105 kDa (Hsp105), accumulate at these foci likely assisting viral particles to escape into the cytosol [27,28]. The exact nature of the penetration foci and the mechanism of translocation are largely unknown. There are no reports on the size of the resulting membrane damage (if any), but the translocated capsid appears to remain intact, therefore requiring the formation of large openings [29]. However, a more recent report suggests that capsid translocation could be coupled to capsid disassembly for genome release, raising the possibility that the inflicted membrane damage is smaller than initially thought [30].

#### 1.1.3. Parvovirus

With a ~25 nm diameter, parvoviruses (e.g., adeno-associated virus (AAV) and canine parvovirus (CPV)) are rather small, non-enveloped DNA viruses that enter cell by endocytosis. Acidification in the endosome is a crucial step to induce conformational changes required for endosome penetration. The drop in pH allows the deployment of the N-terminus of capsid protein VP1, which has a phospholipase type 2 (PLA2) activity [11,31]. This PLA2 activity is essential for endosome penetration, most likely by transient and localized lipid modification [11,32,33]. For example, CPV entry allows the release of 3 kDa dextrans, but not 10 kDa dextrans from endosomes. The absence of co-release of larger molecules during parvovirus endosomal escape suggests that the resulting membrane damage is limited and does not involve complete endosome lysis [31]. However, the exact mechanism of parvovirus membrane translocation is not known.

#### 1.1.4. Reovirus

Reoviruses (e.g., rotavirus) have non-enveloped capsids with a diameter of ~75 nm and contain RNA genomes. The exact entry of reoviruses is unclear, but it is likely to occur through endocytosis. After uptake, endosomal cathepsins are activated by a drop in pH. The outer capsid is then proteolytically processed and capsid protein σ3 is removed [34]. This uncovers the membrane lytic capsid protein µ1 and autoproteolytic processing renders it membrane-lytic [35,36]. The fully processed µ1 N-terminal peptides are then myristylated, released from the capsid and insert into the endosomal membrane, where they form size selective pores [37,38]. While these pores are too small (estimated to 4–9 nm) to permit reovirus translocation, it was suggested that endosome lysis could involve osmotic lysis [39,40].

#### 1.1.5. Picornavirus

Picornaviruses (PV, e.g., polioviruses or rhinoviruses) enter their host cells by endocytosis. Unlike other non-enveloped viruses, PV do not translocate their capsid to the cytosol. Instead, they perforate the endosome membrane by creating a pore to translocate their RNA genome to initiate cytosolic replication [41]. Thus, contrary to viruses like Ad, PV inflicted membrane damage has a small diameter [42]. Receptor binding and a drop in pH provide cues of conformational changes in the virus particle. As a result the amphipathic helix of the capsid protein, VP1 is externalized and VP4 is released to allow membrane binding and pore formation [43,44]. The enzymatic activity of the cellular protein phospholipase A2 group XVI (PLA2G16) then facilitates genome translocation into the cytoplasm, but the exact mechanism of pore formation remains unknown [45]. Some PV encode the 2A protein, which are homologues of the cellular PLA2G16, suggesting an essential and conserved role in membrane penetration [46].

### 1.2. Bacteria Induced Membrane Damage

Pathogen-induced membrane damage has been much more extensively studied in bacterial infections than for viruses. Many intracellular bacteria enter their host cell by phagocytosis, ending up in vacuoles connected with the endo-lysosomal system. To avoid degradation and to proliferate, a number of bacteria escape from their vacuole to the cytosol. Others, such as *Salmonella typhimurium*, first proliferate inside vacuoles and may occasionally escape at a later time point to reach the cytoplasm for hyperproliferation [47]. In all cases, they must destabilize and break the membrane of their vacuole. Numerous studies have shown that this involves bacterial effectors (or their toxins) cooperating with cellular factors. *Shigella flexneri*, for example, is a gram-negative bacteria known to infect epithelial cells. It uses a type 3 secretion system (T3SS) to inject effectors into the cell, resulting in phagocytic uptake. After internalization, *S. flexneri* must escape the vacuole for cytoplasmic proliferation. Initially believed to be mediated via the bacterial T3SS secretion system [48], a subsequent small interfering RNA (siRNA) screening identified the cellular small GTPase Rab11 to be a crucial factor required for vacuole breakdown through the formation of macropinosomes at the invasion site [49,50].

*S. typhimurium* is another bacterial example for membrane damage and probably the best documented. *S. typhimurium* enters its target cells by endocytosis and establishes itself in a replication vacuole (SCV). A small but significant fraction of the bacteria escapes to reach the cytosol, through their T3SS [51]. TANK binding kinase 1 (TBK1) was suspected to control the integrity of the SCV membrane because in the absence of TBK1, *S. typhimurium* replication was more efficient [52]. More recently, it was shown that TBK1 instead coordinates an autophagic response against bacteria after cytosolic exposure (see Section 2) [53,54]. Some evidence suggests that the accumulation of COPII complexes on the SCV membrane destabilizes the membrane through a mechanism that remains to be deciphered [55].

*Listeria monocytogenes* encodes Listeriolysin O (LLO), a toxin it uses for vacuole lysis. Listeriolysin O inserts into the vacuole membrane by binding to cholesterol and forms membrane- disrupting pores. The LLO effect is potentiated by cellular factors including gamma-interferon-inducible lysosomal thiol reductase (GILT) and cystic fibrosis transmembrane conductance regulator (CFTR) [56,57]. Once in the cytoplasm, *L. monocytogenes* replicates and controls the polymerization of actin to be able to propagate from cell-to-cell in secondary vesicles. Unlike the first endosomal escape, escaping from secondary vesicles needs destabilization of two membranes. *L monocytogenes* accomplishes this task using LLO as well as the phospholipases PIcA and PIcB [58,59]. The use of a bacterial toxin for membrane rupture is not unique to *L. monocytogenes* and other bacterial toxins, such as perfringolysin *(Clostridium difficile)*, pneumolysin (*Streptococcus pneumonia*), and VacA (*Helicobacter pylori*) may be deployed from bacteria to cause membrane penetration for the same purpose [60,61,62]. Escape from vacuoles has been demonstrated for several other bacteria including *Mycobacterium tuberculosis*, *Rickettsia prowazekii*, *Burkholderia pseudomallei*, and *Francisella tularensis* [63,64,65,66,67,68,69,70,71,72,73]. The underlying mechanisms are less clear, but generally involve the use of endogenous bacterial genes in conjunction with cellular factors, such as phospholipases, to breach vacuolar membranes. Interestingly for all described examples, these bacterial genes are often associated with pathogenicity linking membrane damage with disease [68].

### 1.3. Membrane Damage Induced by Other Pathogens

Membrane damage is not restricted to bacteria and viruses. There is increasing evidence for a role of membrane damage during fungal and parasite infection. *Candida albicans* is a polymorphic fungus that can change from a yeast form to a hyphal form [74]. After phagocytosis in the form of yeast, conversion into the hyphal form inside the phagosome involves a change in morphology through stretching, which ultimately leads to the rupture of the phagosomal membrane [75]. This is likely to occur through mechanical pressure (filamentation) rather via a biochemical process [76]. As part of a complex life cycle, the protozoa *Trypanosoma cruzi* invades red blood cells and escapes its entry vacuole to reach the cytoplasm [77]. This vacuolar escape depends in part on the parasite PFP TcTOX protein, which seems to have a similar role as the LLO bacterial toxin of *L. monocytogenes.* Additional factors may be involved in breaching the membrane and the precise mechanism needs further clarification.

### 1.4. Non-Pathogenic Membrane Damage

Many neurodegenerative diseases and cancer cells are associated with the induction of sterile membrane damage [78]. In neurodegenerative diseases, membrane damage is often a consequence of the formation of large amyloid aggregates, propagating from cell-to-cell. Studies on α-synuclein showed the ability of these amyloid aggregates to induce endosome rupture after endocytosis [79,80]. This capacity is not restricted to α-synuclein, but also extends to Tau and Huntingtin assemblies [80]. The exact reason why cell invasion by amyloid assemblies and their ability to break the membrane of the endosome causes disease is not fully understood, but it is likely to involve dysregulation of autophagy associated with irreparable damage [80]. Lysosomotropic compounds have emerged to artificially trigger lysosome damage and induce cell death, e.g., as therapeutic concept in cancer [81]. Conceptually, the acidic pH inside makes lysosomes susceptible to the accumulation of weak bases that can penetrate them. Once inside, they get protonated and stay trapped within the lysosome where they can induce specific membrane damage [81]. One compound, the L-leucyl-L-leucine methyl ester (LLOMe), is often used as a positive control in endolysosomal rupture experiments in combination with detection of membrane damage markers [82,83]. Endosomal membrane damage can also be caused via a mechanical/physical trigger, especially in the context of intracellular drug delivery, where it is important to achieve efficient endosomal escape. Some lipid formulations used in transfection of siRNAs were shown to be particularly efficient in causing endosomal membrane damage [84,85]. Gold nanoparticles and nanodiamonds were also employed to physically disrupt the endosomal membrane [86,87].

The non-exhaustive summary of possible pathogenic and non-pathogenic membrane insults presented in this section provides insight into the diversity of threats to cellular membrane integrity. This listing also highlights the importance for cellular surveillance of membrane integrity and the ability to respond to membrane damage to secure cell survival, which is discussed in the next section.

## 2. Membrane Damage Recognition and Cell Response

Extensive damage in endolysosomal vesicles can leak their content and flood the cell with reactive oxygen species (ROS), acid hydrolases (cathepsins), and/or calcium ions provoking injuries to several organelles and ultimately resulting in cell death through necrosis or pyroptosis. A key question, when trying to understand how viruses and other pathogens inflict membrane damage, is how cells sense their membranes have been actively penetrated and how they mount a response. A critical role in the detection of membrane damage is done by cytosolic lectins belonging to the family of Gal. Galectins recognize complex glycans present on lipids or glycosylated proteins. These glycosylated substrates are mainly localized at the cell surface or protruding from the intra-luminal membrane leaflet, including the interior of endosomes and lysosomes. In contrast, they are virtually absent from the cytosol. Hence, membrane damage exposes these glycans to the cytosol and they become easy targets for Gal sensing.

### 2.1. Galectins and Their Role in Membrane Damage Recognition

Galectins are characterized by the presence of one or two carbohydrate recognition domains (CRD) [88]. They can be monovalent (with a single CRD: Gal-1, 2, 5, 7, 10, 11, 13, 14, 15), bivalent (with two CRDs, Gal -4, 6, 8, 9 and 12) or even chimeric (Gal3) [89,90]. The diversity of Gals allows them to have different affinities for a plethora of substrates, making them ideal pathway mediators. Their cellular functions include regulating cell adhesion, organizing membrane domains, signaling and trafficking, apoptosis and cell cycle regulation. Here, we focus on their role as danger sensors since they quickly accumulate on galactosides exposed to the cytosol, which are recognized as danger signals at sites of membrane breach [91,92]. Although mammals have 15 different forms of Gal, to date only Gal1, 3, 8 and 9 have been described to sense damaged vacuoles [2,92]. They accumulate around membranes ruptured by both pathogen and non-pathogen stimuli, such as protein aggregates [80], liposome transfection [85] or lysosomotropic agents [82]. Upon membrane rupture, glycans and Gals form clusters (puncta) that can be easily detected by immunofluorescence using Gal-specific antibodies or cells expressing Gals tagged with a fluorescent protein [82,92,93]. Most Gals (e.g., Gal1/3) are widely expressed, making them useful targets to assess membrane damage [82,93].

Most observations and functional studies using Gals were made using invasive bacteria models. Galectin3 recognizes the vacuoles from which *S. flexneri* escaped and was the first example of a Gal recruited to pathogen-induced membrane damage [92]. A systematic screen including all Gals identified found Gal3, 8, and 9 as binding to *S. typhimurium* ruptured vacuoles [2]. Galectin 8 was also the first Gal shown to restrict bacterial proliferation and thus to have a direct antimicrobial effect [2]. To date, very few studies have addressed if virus membrane penetration also triggers Gal recruitment. Initial studies with Ad used Gal3 as a marker to show the subcellular localization and the timing of Ad endosome penetration [93,94]. In a subsequent study, it was shown that Ad also recruit Gal8 to the endosome penetration site and that this is essential for the cellular autophagy response [3]. Picornavirus pore formation in the endosomal membrane also recruits Gal8-mediating cellular autophagy and remains so far the only other example showing the recruitment of Gals to virus inflicted membrane damage [45]. Whether cells employ Gals in response to entry of other (non-enveloped) viruses remains to be shown. In any case and as discussed below, Ad and PV found ways to counteract Gal8 mediated antiviral autophagy to efficiently propagate.

### 2.2. Galectins and Ubiquitin Coordinate Autophagy for Membrane Damage Removal

The easiest way for a cell to deal with damage is to remove it via degradation pathways. An important part in membrane damage removal has been attributed to autophagy [78]. Autophagy is a conserved cytosolic degradation process induced under stress conditions, such as starvation, hypoxia or during membrane damage and pathogen infection [95], which allows the removal and degradation of damaged organelles or proteins from the cytosol into double membrane vesicles called autophagosomes. These autophagosomes subsequently fuse with lysosomes for cargo degradation and recycling [96]. Autophagy is driven by proteins of the “ATG family” (for autophagy-related), a core set of proteins that coordinates formation, elongation and maturation of autophagosomes [96,97,98]. It is now clearly established that autophagic degradation is highly selective. In mammals, this selectivity is provided by a family of proteins called “autophagic receptors” (including, e.g., the nuclear dot protein 52 kD (NDP52) and p62, reviewed in [99]), which allow targeting of distinct substrates for degradation. Each autophagic degradation process is specified by a “cargo-defined” name such as mitophagy defining the selective degradation of mitochondria, xenophagy for specific degradation of intracellular pathogens or lysophagy naming the degradation of lysosomal membranes upon endolysosomal damage [99].

**Figure 2:**
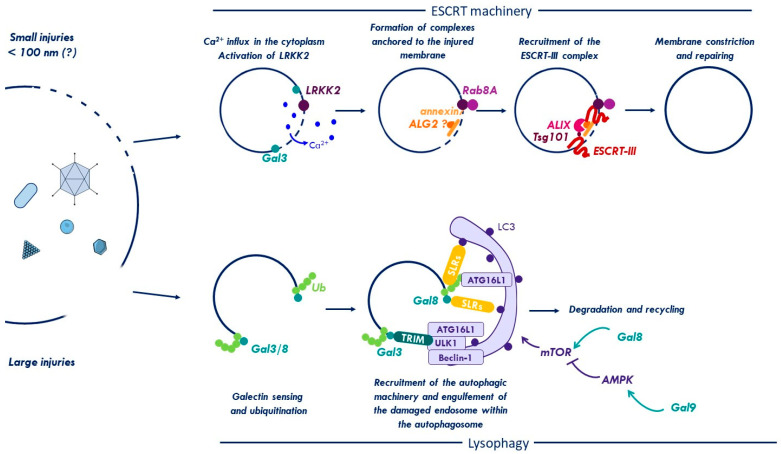
Membrane damage repair and removal. The endosome is constantly challenged by pathogens (bacteria, virus, and fungi), protein aggregates, or chemicals that can disrupt its membrane and provoke injuries of different sizes. Small disruptions (<100 nm) trigger a leakage of Ca^2+^ into the cytoplasm and activate LRKK2. LRKK2 and Ca^2+^ effectors mediate the recruitment of the ESCRT machinery to promote repair of the injured organelle. Galectin-3 (Gal3) is recruited to damage sites and may promote ESCRT assembly. If the injury is too large, the cell will trigger a process of degradation called lysophagy. During lysophagy, damaged vacuoles are sensed and tagged by galectins (Gal) and ubiquitin (Ub). Both signals mediate the recruitment of the autophagic machinery (either directly via autophagic receptors, such as the sequestosome like receptors (SLRs) or indirectly via TRIMs). The membrane remnant is engulfed in a double-membrane vesicle called autophagosome, which fuses with lysosomes for content degradation and recycling. Autophagy is also controlled by metabolic kinase mechanistic target of rapamycin (mTOR) through Gal8. Moreover, Gal9 can also control autophagy induction by directly activating AMP-activated protein kinase (AMPK) in response to endosomal damage to inhibit mTOR. See text for details. Abbreviations: ESCRT, endosomal sorting complexes required for transport; Gal8, galectin8; LRRK2, Leucine-rich repeat kinase 2; ROS, reactive oxygen species; Ub, ubiquitin.

Membrane damage removal by autophagy can be separated into two steps (Figure 2, lysophagy). In a first step, the cell has to sense and discriminate intact vs. damaged vacuoles. The second step is to assemble and recruit the autophagic machinery to degrade membrane remnants. Key elements of the first step are Gals and ubiquitin. Both are so called “eat-me signals”, a name playing with their function to mark membrane remnants for autophagic removal. Certain Gals recognize membrane damage by virtue of complex glycan exposure e.g., during pathogen infection [2,92,93]. To enlist autophagy, Gals have to bind downstream effectors. Thurston et al. reported that Gal8 recruited after SCV rupture forms a complex with the autophagy receptor NDP52 [2]. NDP52 binds FAK family kinase-interacting protein of 200 kDa (FIP200), which serves as seed assembly platform for autophagy initiation [100]. FIP200 assembles a primary kinase complex, the Unc-51 like autophagy activating kinase (ULK1/2) complex, ATG13, and ATG101. Once assembled, this complex activates a downstream Class III PI3-kinase complex with VPS34 and Beclin-1 [100,101]. The Beclin-1 complex modifies membranes and creates a phospholipid patch (also termed phagophore) to support binding of WD-repeat protein interacting with phosphoinositides (WIPI) proteins, creating a membrane anchored landing platform. WIPI proteins recruit the ATG5-ATG12/ATG16L E3 conjugation complex to the phagophore [102]. The ATG5-complex conjugates soluble ATG8 (better known as LC3B) with phosphatidylethanolamine (PE) to be incorporated into the expanding autophagosomal membranes [103]. Additional interactions, such as NDP52-LC3 and FIP200-ATG16L stabilize the developing cargo-autophagosome complex to promote membrane elongation. Gal8 and Gal3 present at the site of membrane damage can also interact with other effectors such as the tripartite motif protein 16 (TRIM16), as shown for ruptured lysosomes using LLOMe or bacteria [104,105]. TRIM16 serves as another assembly hub to initiate autophagy by recruiting the core autophagic machinery (ATG16L1, ULK1, and Beclin-1) directly on damaged membranes [104]. Interestingly, the recruitment of Gal3 to the membrane damage side can also be pro-bacterial by impeding on the autophagic response [106]. Silencing of Gal3 during *L. monocytogenes* infection in murine macrophages increased LC3 recruitment to the vacuoles and reduced bacterial replication. Moreover, treatment of cells with sialidase (which removes sialic acid from glycans) increased Gal3 and decreased Gal8 on the damaged phagosomes. This change in Gal preference directly influenced the autophagic response [106], showing that cytosolic Gals can discriminate the origin of the damaged membrane and orchestrate an adapted cellular response. Deciphering the glycosylation pattern driving Gal specificity could thus be a valuable point for therapeutic intervention. Furthermore, Gal3 also directs other antibacterial functions including the recruitment of interferon (IFN)-inducible guanylate binding proteins (GBPs) to pathogen-containing vacuoles [107].

Next to Gals, ubiquitin is the second most important early danger signal of many membrane damage sites following bacterial [2,108,109,110], viral [3,111], and apathogenic insults [112]. Ubiquitin appears shortly after Gals, and like them, is involved in the recruitment of a variety of autophagic receptors (also known as sequestosome like receptors or SLRs), which contain ubiquitin binding motifs. All SLRs also contain LIR-domains (LC3-interacting region) and bridge the ubiquitylated cargo and LC3 to link the cargo into the growing autophagosomal membrane [99]. Moreover, ubiquitin can directly bind the autophagic machinery through ATG16L1, which may enhance its anchoring to the surface of ubiquitylated endosomes [113]. Two types of ubiquitin chains, K48- and K63 linked, are predominantly found on damaged lysosomes [113]. K63 chains are targeted by SLRs and recruit the autophagy machinery [113,114]. The enzymes driving ubiquitylation of membrane damage, and how the chain type influences the cell response, are not entirely clear, and may involve more than one player. Some of them have been characterized in the context of bacterial infection, where they allow the ubiquitylation of both the bacteria and the membrane remnants to turn them into signaling platforms. The first E3 ubiquitin ligase described to target bacterial membrane damage was the LRR-containing RING E3 ligase (LRSAM) and is required for autophagic clearance of *S. typhimurium* [115]. Another E3 ligase is linear ubiquitin chain assembly complex (LUBAC), which mediates the formation of M1-linked ubiquitin chains and is also involved in autophagic degradation of *S. typhimurium* [116]. The aforementioned TRIM16, recruited via Gal3/8 to endomembrane damage, is also an E3 ubiquitin ligase promoting K63-linkage to ATG proteins and to ubiquitylate lysosome substrates e.g., after LLOMe treatment [104]. FBXO27 (F-Box Protein 27, part of the SCF ubiquitin ligase SKP1/CUL1/F-box protein complex) ubiquitylates several glycoproteins present on the surface of damaged lysosomes (e.g., lysosomal-associated membrane protein LAMP2), which in turn promotes the recruitment of the autophagic machinery [112]. A recent siRNA screen identified a crucial role for Ubiquitin Conjugating Enzyme E2 Q Family Like 1 (UBE2QL1), an E2 ubiquitin conjugating enzyme, in directing lysophagy [117]. Depletion of UBE2QL1 increased steady-state lysosomal membrane damage and prevented efficient ubiquitylation and SLR recruitment, essentially curtailing the autophagy response. It also prevented recruitment of the AAA-ATPase valosin-containing protein, VCP/p97 complex. The VCP/p97 complex is likely to extract membrane proteins to facilitate downstream lysophagy and cooperates with YOD1/OTU1, a K48-specific deubiquitylating enzyme. This observation suggests that selective membrane deubiquitylation, possibly of K48 labeled VCP/p97 substrates, maybe necessary before lysophagy proceeds [78,118]. A similar mechanism appears active in neurodegenerative diseases, where protein aggregates rupture endo-lysosomes and Gal3/8 recruits NDP52 and p62 suggesting that the response might be cell intrinsic [80,119].

Some receptors including NDP52 and p62 are phosphorylated by TBK1, to enhance their ability to bind ubiquitin following recruitment to the membrane damage site [120,121]. TBK1 also phosphorylates WIPI proteins and enhances autophagosome formation in response to bacterial membrane penetration [54,122]. Thus, TBK1 plays an important role in promoting and fine-tuning the antimicrobial autophagy response at the site of membrane damage.

On a larger scale, autophagy is under the control of two different kinases, the metabolic Ser/Thr kinases mechanistic target of rapamycin (mTOR) and AMP-activated protein kinase (AMPK). Both kinases are major regulators of autophagy. They act in an antagonistic way in that mTOR suppresses autophagy by phosphorylating inhibitory sites on autophagy regulators, such as ULK1/2, while phosphorylation by AMPK activates autophagy regulators [123]. Active mTOR can be found on the outer lysosomal membrane and upon lysosome damage translocates to the cytosol and becomes inactivated. Keeping mTOR inactive involves inhibitory phosphorylation by AMPK, connecting both kinases. It was recently shown that Gal8/9 regulate mTOR and AMPK on damaged lysosomes identifying new functions beyond glycan recognition [4]. The study shows that interaction of Gal8 with the lysosome membrane integral neutral amino acid transporter Solute Carrier Family 38 Member 9 (SLC38A9) upon membrane damage reorganizes a signaling complex (the Ragulator-Rag complex) on the lysosomal membrane. The Ragulator-Rag complex normally retains mTOR in an active state bound to the lysosome surface due to nutrient sensing. The reorganization through Gal8 (e.g., following membrane damage) promotes mTOR dissociation from lysosomes. Inactivation of mTOR is then reinforced by the action of another Gal, Gal9, which activates AMPK [4]. Taken together, this work offers the interesting perspective that membrane damage can be controlled by Gals at two different levels. Locally, at the physical site of membrane damage through recruitment of SLRs and assembly of the autophagic machinery. In addition, Gals also act at the cellular level by coordinating the activities of mTOR and AMPK. This hierarchical organization could be used to amplify and disseminate the local response, e.g., dependent on the extent of the damage by modifying major upstream autophagy regulators. Widening the response via mTOR inactivation has several effects on the cell, including the nuclear translocation of transcription factor EB (TFEB), a mTOR target, and master regulator normally sequestered in the cytosol, which drives gene expression programs for neosynthesis of genes involved in the biogenesis of lysosomes [104], in autophagy [124], and in inflammation [125].

### 2.3. Viral Control of the Autophagy Response to Membrane Damage Removal

**Figure 3:**
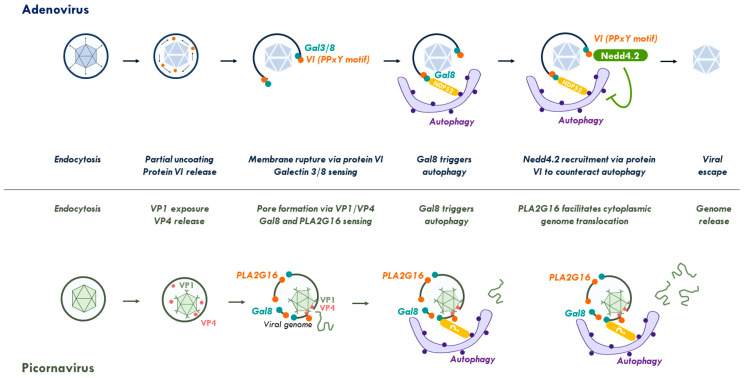
Viral control of the membrane damage response. Top: endocytosed adenoviruses partially uncoat and release the membrane lytic capsid protein VI for endosomal membrane lysis. Membrane damage is sensed by galectins 3 and 8. Galectin 8 recruits autophagic receptors and triggers autophagy. Adenoviruses stall autophagy through a short PPxY peptide motif in protein VI that recruits the ubiquitin ligase Nedd4.2. As a consequence, they avoid degradation and escape into the cytoplasm. Bottom: After endocytosis and acidification of the endosome, picornaviruses undergo conformational changes to expose capsid protein VP1 and release of VP4. Both proteins attach to the endosomal membrane creating membrane-penetrating pores. Membrane damage is then independently sensed by galectin 8 activating autophagy and PLA2G16. PLA2G16 facilitates genome translocation into the cytoplasm preventing autophagic clearance. See Section 2 and Section 3 for further details. Abbreviations: Gal, galectin; NDP52, Nuclear dot protein 52; Nedd4.2, neural precursor cell expressed, developmentally down-regulated 4.2; PLA2G16, phospholipase A2 group XVI.

Most of the investigations concerning the cellular response to membrane damage use invasive bacteria or models of induced lysosome damage. Although details are still emerging, there seems to be consistency since cells respond in all cases with autophagy directed against the membrane damage site. Viral membrane damage is no exception, although only two viral systems have been investigated. Adenovirus and PV both cause different types of membrane damage upon entry (Figure 3). Depending on the cell system used, Ads penetrate the endosome within 15–20 min and trigger fast selective autophagy in the infected cell. Adenovirus damaged endosomal membranes are detected by Gal3 [93,94,111] and Gal8 [3]. Using co-detection of viral particles and/or the exposed membrane lytic protein VI revealed that the viral association with ruptured membrane is transient, and that the Gal3/8-positive membrane associated with NDP52, p62, ubiquitin and LC3 [3,111]. Gal3 positive membrane remnants were cleared from the cell within 3 h of entry presumably via autophagy [111]. Interestingly wild type Ad was not cleared by autophagy and suppressing autophagy with genetic or pharmacological tools did not affect viral infectivity [3]. Using the mutant Ad-M1, the authors showed that being refractive to autophagic clearance is not simply due to a rapid escape from the endosome, but also involves active control of autophagosome maturation. The mutant Ad-M1 has strongly reduced infectivity compared to wild type Ad. It lacks a PPxY motif encoded in the membrane lytic protein VI, which is required to recruit the ubiquitin ligase neural precursor cell expressed developmentally down-regulated 4-like (NEDD4.2) upon membrane penetration [18]. Unlike wild type Ad, Ad-M1 is unable to escape from endosomes and becomes susceptible to autophagic degradation [3]. Comparing both viruses revealed that wild type viruses do not prevent autophagy initiation. Instead, they use the PPxY motif to prevent autophagosomes from maturing and fusing to lysosomes, a process that was also observed upon NEDD4.2 depletion from cells using viral and non-viral autophagy stimuli [3]. The exact role for NEDD4.2 is not yet clear and may involve regulating autophagy effectors such as ULK1 or protein VI itself [18,126]. However, this example shows how Ad has evolved a short peptide motif that recruits an essential cellular factor to stall the cell response to membrane damage, thereby buying enough time to reach the safety of the cytosol. The PPxY motif is strategically located in the membrane lytic factor making sure it is exposed at the right time and place. Consequently, eliminating membrane damage recognition by depleting either Gal8 or autophagy (e.g., in ATG5 KO cells), fully restores viral infectivity of the PPxY-deficient Ad-M1. In contrast, this does not accelerate viral escape from the endosome, showing that escape mechanism and autophagy suppression are separate processes, and that one favors the other [3].

Unlike Ad, which generates large openings in the endosomal membrane, PV creates pores of limited size within endosomes to translocate their RNA genome across the membrane. A haploid cell-based genetic screen identified the small phospholipase PLA2G16 (Group 16 phospholipase A2) as an essential factor required for genome translocation [45]. Using a counter screen in PLA2G16 deficient cells, the authors found that depletion of several autophagy genes (e.g., ATG5/7/12) and Gal8 restored viral infectivity. Using fluorescent viruses and alternative membrane rupture assays, they could show that Gal8 and PLA2G16 relocated after membrane damage independently of each other. This suggests that PLA2G16, like Gal8, senses virus-induced membrane rupture, but is recruited in a virus- and Gal8-independent manner. PLA2G16 has no effect on the autophagic flux itself, but facilitates the translocation of the genome into the cytoplasm and prevents its clearance by autophagy. When PLA2G16 is silenced, genomes are degraded via autophagic clearance together with the virus, a phenotype that can be reversed if Gal8 or ATG7 is also silenced [45]. This work thus identified PLA2G16 as cellular factor exploited by a virus to control antiviral autophagy directed against PV-induced membrane damage, a strategy analogous to the PPxY motif of Ad. Unlike for Ads, PV protection from autophagic degradation through accelerated cytosolic translocation concerns only the viral genome. It is conceivable that the enzymatic activity of PLA2G16 favors PV genome translocation over autophagic clearance of the membrane damage by local lipid modification, although the exact mode of action is currently unclear. Interesting in both cases, neither Ad nor PV have evolved mechanisms to suppress autophagy initiation suggesting that there is additional benefit in triggering autophagy, possibly linked to counteracting inflammatory signaling (Section 4).

### 2.4. ESCRT Machinery for Membrane Repair

Cells use autophagy to remove and degrade damaged membranes such as lysosomes (lysophagy) for cell survival [2,104,112,113]. However, the cell response can be more restrained and small membrane injuries were shown to trigger a membrane repair mechanism via the ESCRT system rather than removal [127,128,129] (Figure 2, ESCRT machinery). The ESCRT complex is divided into five sub-complexes (ESCRT-0, I, II, III, and disassembly proteins) and it was first discovered for its role in regulating endosomal trafficking, but is now known to be involved in numerous other cellular processes such as vesicle formation, vesicle budding and cytokinesis as detailed elsewhere [5,130]. Small membrane lesions (<100 nm), especially at the plasma membrane, trigger calcium ion influx and lead to a rapid recruitment of cytosolic annexin7 and the calcium sensor ALG2 at the membrane damage site. This complex recruits subsequently the ALG2 interactors ALIX and Tsg101, both ESCRT-I proteins that orchestrate the recruitment of the ESCRT-III complex to repair the membrane. ESCRT-III has the ability to form filaments that constrict the membrane and shed the damaged membrane [5,127,128]. A recent report showed that the ESCRT machinery was also quickly recruited to damaged endo-lysosomes to allow their repair following treatment with either LLOMe or silica crystals [131]. Even if the initial recruitment of ALG2 (apoptosis-linked gene-2) is still debated [129,131], endosome repair requires recruitment of the ESCRT-I components ALIX (ALG-2-interacting protein X) and Tsg101 (tumor-susceptibility gene 101), and the subsequent action of the ESCRT-III complex. Repair also involves transient inactivation of lysosomal hydrolases followed by re-acidification [132]. Even with minor damage, Gal3 is still recruited to damaged endo-lysosomes, but follows a slower kinetic than ESCRT recruitment. The ESCRT-III response (monitored by recruitment of CHMP4B/ESCRT-III) occurs on the timescale of minutes preceding lysophagy that mounts within the hour [129,131]. Depletion of either ALIX or Tsg101 or both does not affect Gal3 recruitment, but delays membrane repair in favor of removal/lysophagy [129,131]. A partial explanation for this observation might be that Gal3 initially promotes interactions between ALIX and the downstream ESCRT-III effector CHMP4. At later times, however, Gal3 controls the autophagic response via TRIM16 supported by Gal8/9 that regulate mTOR/AMPK [133]. A very recent study showed that macrophages challenged with either invasive bacteria or LLOMe activates the Parkinson’s disease related kinase leucine-rich repeat kinase 2 (LRKK2), which in turn recruits the Rab GTPase Rab8A. Both coordinate the activity of the ESCRT-III complex for membrane repair. In contrast, depletion of LRKK2 and Rab8A change the damage response phenotype from membrane repair to lysophagy [134].

The interplay of Gal3/8/9 with the ESCRT machinery, autophagy and metabolic signaling is also observed during membrane damage upon *M. tuberculosis* and *Coxiella burnetii* infection [129,133]. Interestingly, mycobacterial effectors EsxG/TB9.8 and EsxH/TB10.4 secreted by the ESX-3 T7SS secretion system antagonize the ESCRT response with a kinetic that matches the speed with which the cell responds, showing that bacteria have also developed efficient countermeasures against membrane repair mechanisms [135]. It is still possible that ESCRT and autophagy have overlapping roles in endosome repair because ATG5 was shown to be involved in repairing the endosomal membrane damaged by the type-1 secretion system T1SS after infection with *S. typhimurium* [136]. ESCRT and autophagy also cooperate in the maintenance of the bacterial vacuole in *Dictyostelium discoideum* infected with *Mycobacterium marinum* [137]. Taken together the cell reaction to membrane damage appears conserved, fast, and flexible. This implies an active and hierarchical organization, where the ESCRT-mediated membrane repair is transient and precedes lysophagy [129,133].

### 2.5. Viral Control of the ESCRT-Response to Membrane Repair

If there is an ESCRT-response against membrane penetration by non-enveloped viruses upon entry has not been investigated. The ESCRT machinery plays a major role in the release process of most enveloped viruses and mediates their non-lytic release from cells (including some non-enveloped viruses). Many viruses use a conserved PPxY peptide motif or the related P(S/T)AP motif encoded in their capsid proteins to recruit ESCRT-I proteins or NEDD4 ubiquitin ligases (for review see [138,139,140]). These domains are still present in entering virus particles and could easily target the ESCRT machinery during entry. As discussed above, such a conserved PPxY domain able to recruit NEDD4-family ubiquitin ligases is encoded in the Ad membrane lytic protein VI [18], which suppress Gal8-mediated antiviral autophagy upon endosome penetration likely through a NEDD4.2 mediated process [3]. It is not clear if this is functionally related to the ESCRT machinery. A different study showed that upstream of endosomal escape, the binding of Ad to the cell surface results in small (transient) lesions at the plasma membrane in a protein VI-dependent manner that provokes extracellular calcium ion influx and dye penetration into the cell [128]. This local increase in calcium ions triggers exocytosis and local fusion of secretory lysosomes as part of a repair mechanism [128]. Upon membrane fusion, these lysosomes are suggested to release lipid converting acidic sphingomyelinase (ASM) catalyzing sphingomyelin thereby generating high local concentrations of ceramides. Ceramides, when present in the endosomal membrane, increase the affinity of protein VI, helping Ad to penetrate the endosome [19]. Such a mechanism suggests that Ad membrane penetration is a two-step process involving small and large sized membrane damage [19]. As shown recently, sphingomyelins play an important role in initiating the membrane collapse in bacterial vacuoles preceding Gal8 recruitment to exposed glycans [141]. It would be interesting to know if the small lesions observed for Ad in addition to the exocytosis of lysosomes have a similar function and also trigger an ESCRT-response similar to the one described above [127,128]. In this case, capsid-encoded late domains, such as the PPxY motif in protein VI or perhaps a second motif present in the viral penton protein [142], may exert additional control functions upstream of viral restriction of Gal8-mediated antiviral autophagy in response to endosome damage [3]. More generally, if the ESCRT-response directed against small lesions in endosomes or lysosomes is a cell intrinsic response, several other viruses would have to face and counteract such a response.

### 2.6. Inflammation to Signal Membrane Damage

**Figure 4:**
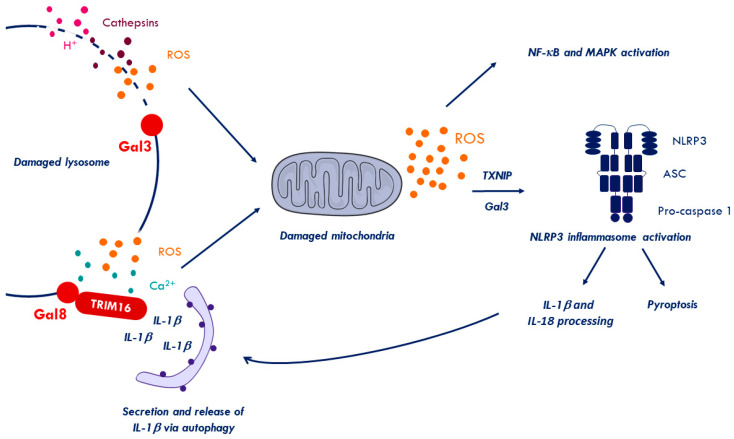
Membrane damage signaling. Endolysosomal rupture triggers the leakage of harmful components (e.g., ROS, H^+^, cathepsins or Ca^2+^), resulting in mitochondrial damage and increased ROS production. Increasing levels of ROS activate nuclear factor-kappa B (NF-κB) and mitogen-activated protein kinase (MAPK) signaling and triggers the release of the thioredoxin-interacting protein (TXNIP) from thioredoxin. Soluble TXNIP mediates Nod-like receptor family, pyrin domain-containing 3 (NLRP3)-inflammasomes activation and association with their adapters (ASC), thereby triggering pro-caspase conversion [149,150]. Activated inflammasomes process pro-inflammatory cytokines (IL-1β and IL-18) and may trigger cell death through pyroptosis. Processed IL-1β in turn can be recruited by Gal8 and TRIM16, that coordinate the autophagic machinery to secrete IL-1β via an unconventional secretory pathway [105,151]. Abbreviations: Gal3, galectin3; Gal8, galectin8; ROS, reactive oxygen species.

Membrane damage, especially in the endo-lysosomal compartment, can be repaired or removed. However, it also enables leakage of soluble contents from the endocytic compartments into the cytosol including protons, ROS, calcium ions, and soluble acid hydrolases such as Cathepsin B. This leakage eventually damages mitochondria further increasing ROS production, which can trigger inflammasome activation [143,144] (Figure 4). Inflammasomes are important cytosolic signaling platform, mediating caspase-1 activation and the secretion of pro-inflammatory cytokines including IL-1β and IL-18 [145]. The best-studied inflammasome is the Nod-like receptor family, pyrin domain-containing 3 (NLRP3)-inflammasome, which can be activated by multiple stimuli, including membrane damage (Figure 4). Its activation can result in cell death by pyroptosis unless cell survival pathways, such as autophagy are activated [146,147]. Not surprisingly, pro-inflammatory signaling and autophagy are an important part of the cellular stress response and are partially overlapping (summarized in [148]).

Recognizing invading pathogens involves pathogen-associated molecular patterns (PAMPs) intrinsic to the invading microbes and damage-associated molecular patterns (DAMPs). DAMPs are best described as out-of-place detection of cell components due to pathogens or stress. The understanding of pro-inflammatory signaling responding to pathogen-induced membrane damage is limited, partly because it is difficult to discriminate membrane damage signaling from microbial PAMP-activated signals. The latter trigger pathogen recognition receptors including lumenal Toll-like receptors (TLRs), cytosolic nucleic acid sensing receptors such as Cyclic GMP-AMP synthase/Stimulator of interferon genes protein (cGAS/STING), retinoic acid-inducible gene I (RIG-I) like receptors (RLRs) and Nod-like receptors (NLRs). The different receptors exert their pathogen sensing role in association with the endocytic entry compartment [152]. To complicate matters even further, different sensing pathways can converge in overlapping responses such as IFN or pro-inflammatory cytokine expression [153]. As discussed above, cytosolic glycan exposure is considered an important and unique DAMP for membrane damage. Thus, inflammatory responses upon non-microbial membrane damage provides some insight into cause and consequences and can be studied e.g., in the context of neuro-degenerative diseases and neuroinflammation [154]. Some, neurodegenerative diseases are caused by the formation of pathogenic protein aggregates. These include amyloid-β or tau in Alzheimer’s disease, α-synuclein in Parkinson disease or aberrant polyglutamine stretches in mutated huntingtin in Huntington’s disease (reviewed in [155]). During cell-to-cell transmission, aggregates reach the cytosol by damaging the endocytic compartment, which results in Gal3 accumulation on endosomes but mostly lysosomes [80]. Furthermore, α-synuclein mediated lysosome rupture produces ROS e.g., due to mitochondrial damage upon cathepsin release [79,149]. Unbalanced ROS accumulating in the cytosol can trigger the inflammasome, but also other inflammatory pathways including the nuclear factor-kappa B (NF-κB) pathway ([156,157], reviewed in [158]). Thus, it is important to understand which pathway is activated upon membrane breach. A recent study using several Huntington’s disease models showed a role for Gal3 in the regulation of the inflammatory response towards membrane damage caused by the huntingtin mutant. Pharmacological inhibition or depletion of Gal3 in respective microglia cells or a mouse model attenuated pro-inflammatory cytokines including interleukins (IL-1β, IL-6) and TNFα. This reduction correlated with reduced NLRP3 inflammasome levels suggesting a direct regulation via Gal3 [159]. Mechanistically, the authors discuss a role for Gal3 in inhibiting the clearing of damaged lysosomes and a possible, Gal3-mediated crosstalk with the NF-κB pathway [160]. Based on these results, it is possible that Gal3 plays part in maintaining the inflammatory signaling upon membrane damage by keeping the inflammasome activated until overruled by autophagy (see above). Other Gals, such as Gal8/9, also contribute to inflammation upon membrane damage. A recent study showed that Gal8 recruited to damaged lysosomes binds to TRIM16, which sequesters processed IL-1β (Figure 4). TRIM16 coordinates autophagosome assembly and transfers IL-1β to secretory autophagic vesicles to enhance IL-1β secretion [105,145]. If Gal3/Trim16 has a similar role is not known. These examples indicate that Gals may have a dual role by regulating inflammatory responses on top off membrane damage control.

### 2.7. Inflammation upon Virus Membrane Penetration

Detecting viral nucleic acids such as double stranded RNA or cytosolic DNA is crucial for cells to sense invading viruses. Toll-like receptors are the first line of sensors (10 in humans) and can detect different danger signals including nucleic acids (DNA and RNA) [161]. They localize either to the plasma membrane (TLR1/2/4/5/6/10) or inside the endosomal compartment (TLR3/7/8/9) [162]. Pathogen-associated molecular pattern detection induces a change in TLR conformation, allowing interaction with adapter molecules, such as Myeloid differentiation primary response protein (Myd88) or TIR Domain-Containing Adapter-Inducing Beta Interferon (TRIF), which activate in turn IRF transcription factors and induce IFN-I production. The cytosolic presence of short double-stranded RNA with a 5′ triphosphate end activates RIG-I [163,164], while long double-stranded RNA activates the Melanoma differentiation-associated protein 5 (MDA5) [165]. These two RLRs recruit MAVS adapter molecules located on the mitochondrial surface were they activate signaling kinases inhibitor of nuclear factor kappa-B kinase subunit alpha (IKKα) and TBK1 [166,167]. Both kinases phosphorylate transcription factors IRF3/7 and NF-κB, which drive expression of inflammatory genes and interferons [167,168]. Reovirus and PV, with their RNA genomes, were shown to activate the RIG-I/MDA5 pathway although probably not during entry [169,170,171,172,173]. In addition, the encephalomyocarditis virus viroporin 2B was shown to activate the NLRP3 inflammasome and to trigger IL-1β release [174,175]. Other PV like human rhinovirus (HRV) or expression of the HRV 2B protein also activated the NLRP3 inflammasome. However, because UV-inactivation of PV strongly decreased inflammasome activation it is unlikely that the PV membrane penetration step plays a dominant role [176]. Moreover, cGAS is a ubiquitous sensor of cytosolic DNA, which recognizes nucleic acids e.g., from entering DNA viruses. In the presence of DNA, it catalyzes the local formation of cGAMP, which binds to the ER associated adapter protein STING. This induces a conformational change in STING, which will then translocate to the Golgi where it serves as phosphorylation platform by recruiting TBK1 and its IRF3 substrate, again resulting in expression of pro-inflammatory cytokines and IFN. The Ad *ts1* mutant was instrumental in identifying inflammatory signaling linked to Ad membrane penetration. As discussed above, Ad *ts1* is endocytosed, but does not rupture the endosome. Wild type virus (but not *ts1*) induces a pro-inflammatory response upon cell entry, characterized by increasing the activity of p38 and Extracellular signal-regulated kinase (ERK) [177]. Likewise, rupture of the endosomal membrane with high doses of wild type Ad in murine macrophages induced the expression of IFN via activation of TBK1/IRF3 in a STING dependent manner [178,179]. Immune-complexed Ad in monocyte-derived DC (MoDC) was shown to release protein VI and to accumulate Gal3 indicative of membrane rupture and to activate the absent in melanoma 2 (AIM2) inflammasome [180]. Using a macrophage model, it was shown that high doses of Ad leads to the rapid production of ROS, release of cathepsins into the cytosol and mitochondrial damage. This resulted in secretion of IL-1β following activation of the inflammasome NLRP3. In their studies neither the Ad-*ts1* mutant nor reovirus was capable of inducing this kind of response [181,182,183]. The data show that inflammasome activation by Ads requires membrane damage and cathepsin release. However, endosome rupture and cytosolic accumulation also exposes the viral DNA to cGAS/STING and RIG-I detection triggering additional immune responses [184,185,186]. The immune activation mechanisms induced by Ad membrane rupture are therefore not easy to dissect and it remains to be established if there is signaling hierarchy or cooperativity. Further evidence of membrane modulation in the direct activation of the antiviral response have also been found in enveloped viruses discussed elsewhere [187,188].

## 3. Crosstalk Between Membrane Damage, Autophagy, and Inflammatory Response

As discussed in the previous sections, membrane damage induces autophagy and inflammation. It is probably fair to assume that autophagy and inflammation elicit some kind of retro-control on each other. Interestingly, several of the inflammatory pathways that are activated upon membrane damage or upon virus entry also result in a net activation of autophagy. Not surprisingly, autophagy was suggested to be involved in regulating and limiting innate and adaptive immunity (reviewed in [189]). An early indication of the direct link between autophagy and inflammation was shown by studying Crohn’s disease in ATG16L1 deficient mice [190]. These mice had high levels of IL-1β and IL-18, which are indicative of elevated inflammasome activation. Similarly, IL-1β production was increased in macrophages treated with the autophagy inhibitor 3-MA or macrophages deficient for several ATGs [190,191]. The absence of ATG7 [192] or ATG5 [193,194] also caused an increase in IL-1β production in murine (alveolar) macrophages and induced pyroptosis showing how important autophagy is in limiting inflammation at least in immune cells. Mechanistically autophagy either removes inflammasome activators, inflammasome substrates or removes the activated inflammasome itself. For example, autophagy selectively degrades damaged lysosomes (lysophagy) to remove a source of cytosolic cathepsins, which can cause depolarization of mitochondria (Figure 4). Damaged mitochondria produce large amounts of ROS or oxidized mitochondrial DNA, both strong inflammasome activators. The selective removal of damaged mitochondria (mitophagy), thus, further restricts inflammasome activity [143,194]. Like lysophagy, mitophagy is a process of selective autophagy and uses selective autophagy receptors such as p62 [195] or Fanconi anemia complementation group C (FANCC) [196]. These receptors are recruited to damaged mitochondria by virtue of ubiquitin tags placed by the ubiquitin ligase Parkin [197] described in detail elsewhere [198]. Autophagy also sequesters and removes the immature form of pro-inflammatory IL-1β, an inflammasome substrate [199]. Autophagy also directly degrades activated inflammasomes. For example, the AIM2 inflammasome is ubiquitylated via the TRIM11 E3 ligase upon activation. The SLR, p62 recognizes the ubiquitin signal on AIM2 and mediates selective autophagy degradation [200,201]. A different TRIM, TRIM20, was shown to ubiquitylate pro-caspase 1 as well as the NLRP1/3 receptor [202], suggesting a more universal degradation mechanism of activated immune regulators by TRIMs (reviewed in [203]). In contrast, several inflammasome receptors interact with Beclin-1 and block the induction of autophagy [204]. This observation suggests that NLR receptors may have reciprocal regulatory functions through direct interactions with ATGs controlling autophagy and inflammasome activation [204]. Autophagic processes are not systematically anti-inflammatory. In macrophages and probably other immune cells, Gal8/TRIM16 sequestration of mature IL-1β to autophagic membranes regulates unconventional secretion of IL-1β [205,206]. Consequently, inhibiting autophagy decreased inflammation in response to activators of AIM2 and NLRP3 inflammasomes in some studies [205,207].

Inflammasomes are not the only signaling platforms degraded by autophagy. Virus sensing in the endosome via TLR, cytosolic RIG-I/MDA5 sensing of viral RNAs or cGAS/STING sensing of viral DNAs all assemble specific signaling platforms that activate interferon and/or proinflammatory cytokine expression. Whether some (or all) of these pathogen sensing and signaling platforms are linked to the surveillance of membrane integrity is not clear. It would make sense for cells to aim to understand what penetrates the membrane and establish some mechanistic link between membrane damage detection, sensing, signaling, and removal by integrating autophagic and immunity signaling. This idea is supported by several observations. Autophagy can be induced following the activation of certain TLRs [208,209]. For example, TLR7 and 3 (involved in innate responses to viruses) induce autophagy in vitro after their activation by single-stranded and double-stranded RNA present in viral genomes [210,211]. The precise mechanism is not known, but involves recruitment of Beclin-1 [211]. Autophagy in turn limits TLR signaling by degrading several mediators such as IKK/TBK1 and IRF3/7 (see below, [203]). ATGs (and autophagy) are also involved in preventing unprovoked RIG-I pathway activation by blocking the interaction between RIG-I and mitochondrial antiviral-signaling protein (MAVS). For example, ATG12-ATG5 inhibits RIG-I and MAVS by binding to their caspase activation and recruitment (CARD) domain [212]. A similar function was shown for Beclin-1 blocking RIG-I/MAVS also by binding the CARD domain of MAVS [213]. In contrast, once activated, RIG-I/MAVS trigger the production of IFN and the expression of interferon inducible genes (ISGs). Two ISGs, tetherin and ISG-15, provide a negative feedback loop targeting respectively MAVS and RIG-I for selective autophagy [213,214,215]. Autophagic restriction of cGAS/STING works in a similar fashion. ATG9 prevents activation of TBK1 by controlling STING [216], while Beclin-1 binds cGAS to prevent the production of cGAMP and thereby IFN production [217]. In addition, at steady state, cGAS is rapidly turned over via K48 ubiquitylation, recognized by p62 and targeted to selective autophagy [218]. This constant turnover is counteracted upon cGAS stimulation by the ISG TRIM14 [218]. STING can also be ubiquitylated presumably by TRIM56 [219] resulting, like for cGAS, in degradation via p62 and selective autophagy [220]. Both pathways, RIG-I/MDA5 and cGAS/STING recruit kinases TBK1 and IKK respectively to phosphorylate transcription factor IRF3/7. Like the different signaling platforms (including the inflammasome), the kinases and transcription factors substrates are also subject to autophagic control. TRIM21 is capable of recruiting the autophagic machinery and initiating de novo autophagy to target IKKβ [221], while TBK1 is degraded via a TRIM27 mediated process [222]. In addition, TRIM21 controls autophagy degradation of active IRF3 dimers [202]. This by far non-exhaustive list of examples shows how intricate and tight innate immunity and the autophagic degradation machinery work together to exacerbate and restrict the inflammatory response.

## 4. Concluding Remarks: What Is in It for the Virus?

Our understanding of the cellular membrane damage response has much increased over the years. Most of our knowledge stems from membrane damage caused by invasive bacteria or by mechanical disruption through protein aggregates or lysosomal damage. With the study of virus-induced membrane damage, the field grows further and it appears that the cell response is conserved and linked to pro-inflammatory signaling.

Whatever the trigger, cells seem to be able to determine the size and extend of membrane damage. The first membrane damage response “repair me if you can” involves the ESCRT machinery, while larger and sustained damage then triggers autophagy and probably pro-inflammatory signaling. As summarized in this review, Gals, SLRs, ATGs, and TRIMs are four interconnected protein families at the heart of the cellular response to membrane damage, regulating autophagy and controlling the ensuing inflammatory response [148,203]. Galectins detect the membrane damage and guide the ATGs via SLRs to start the autophagic process. So far, only one TRIM, TRIM16, was demonstrated to directly participate in the cell response to membrane damage driven by Gals and autophagy. However, more than half of > 60 TRIMs screened in two independent approaches were found to be positive regulators of autophagy ([223,224]) or virophagy (e.g., TRIM21/23/41) ([223]). In addition, a plethora of articles show that several TRIMs participate in innate immunity, either by regulating the TLRs (e.g., TRIM8/30a/31/32/38/56) and/or the nucleic acid sensors RIG-1/MDA5 and cGAS/STING (e.g., TRIM13/14/25/29/31/32/38/56) or by directly targeting viruses (TRIM 5α/21/22). The respective TRIM targets are almost always subjected to autophagic degradation (reviewed in [203] and references there in). Interestingly, a variety of the TRIMs involved in immune or autophagy regulation were also shown to bind Gal3/8 in a pull down assay (TRIM5α/6/17/20/22/23) [104]. To date, it is not known if Gal binding and autophagy or immune regulation by these TRIMs functionally bridges membrane damage recognition with immune regulation and/or pathogen sensing.

**Figure 5:**
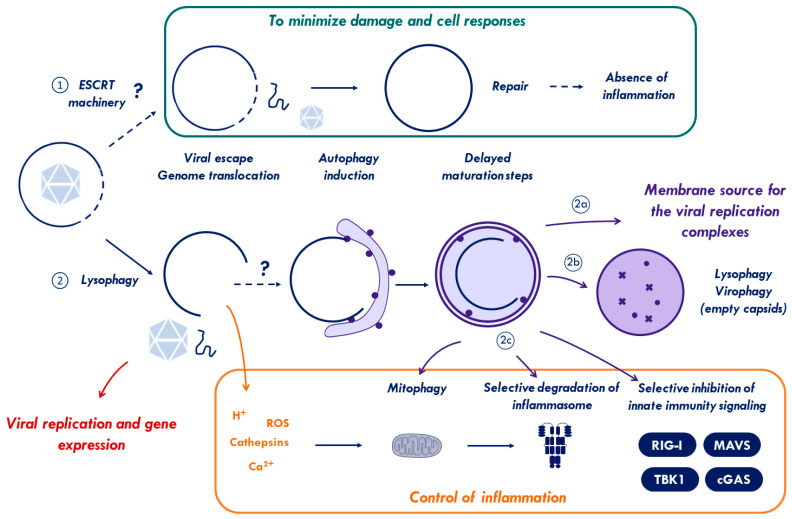
Model for non-enveloped virus membrane penetration. Non-enveloped viruses breach endo-lysosomal membranes for particle escape into the cytosol or cytosolic genome translocation. (1) If the membrane damage is small enough, Ca^2+^-dependent recruitment of the ESCRT machinery will repair the endosomes limiting time and extend of the damage. This strategy could provide enough time for the virus/genome to reach the cytosol without activating the inflammatory pathways. (2) If this repair fails or the inflicted damage is too large, “eat-me signals” (galectins and ubiquitin) will accumulate at the damage site and initiate autophagy. Viruses use viral factors (e.g., Ad) or cellular factors (e.g., PV) to delay the autophagy response until capsids/viral genomes reach the safety of the cytosol. The ensuing autophagy response than may continue and work in favor of the virus. Activating autophagy via membrane damage could (2a) provide a membranes source for viral replication, and (2b) remove and recycle membrane remnants, damaged lysosomes, and empty viral capsids to avoid excessive cells responses. Additionally, autophagy could (2c) limit excessive inflammation through autophagic degradation of damaged mitochondria and by removing activated inflammasomes and effectors of the innate immunity signaling pathways (see text for details).

Very few data exist today concerning viral membrane damage especially elicited by non-enveloped viruses during cell entry. One particularity of virus entry is that unlike bacteria, entering viruses are unable to express and use effector molecules against the cell defenses until their genomes are delivered and expressed. Consequently, viral capsid components are the only available means to counteract the cell response. The two examples discussed in this review, Ad and PV, have in common that they stall the membrane damage response by delaying autophagy until they delivered either the capsid (Ad) or their genome (PV) to the cytosol. Moreover, both viruses induce a cellular membrane damage response as shown by using a mutant virus or depletion studies suggesting some advantage connected to the cell response during virus entry. This is an important observation because membrane damage or antiviral autophagy can be deleterious for the virus. For example, following uptake into human macrophages, Ad particles opsonized with antibodies were shown to rupture lysosomes and to trigger inflammasomes, while cytosolic TRIM21 would recognize any opsonized virus that made it to the cytosol and hand it over to cGAS/STING sensing [225]. As discussed above, inflammasome and cGAS/STING are down regulated via autophagy. Likewise, Ad-M1 mutant viruses are unable to stall the membrane damage response upon entry because they lack an essential PPxY peptide motif. As end result, Ad-M1 is degraded via autophagy and presented much more efficiently to MHC class II than wild type Ad in a murine model [3]. Furthermore, during the cell response elicited by bacteria, TBK1 phosphorylates SLRs independent of its IFN promoting role via IRF3 phosphorylation [122]. Without delaying autophagy, PV genomes are degraded with their capsids [45]. In contrast, it is well known that PV exploit autophagy to create a membrane compartment for cytosolic genome replication (reviewed in [226]). Delayed autophagy activation upon genome translocation therefore may facilitate the onset of PV replication. To put this into a more general perspective, it appears possible that non-enveloped viruses actively use the cellular membrane damage response during entry to limit the cell intrinsic immune response (Figure 5). Some viruses may have evolved to escape the endosome using openings that are small enough to be repaired inflicting only local and transient damage without significant immune activation. The opposite scenario with activating autophagy through extended membrane damage and controlling it (like Ad and PV), thus may come as a trade-off. On one hand, it allows endosomal escape and the exploitation of autophagic membranes for replication while autophagy maybe used to accelerate the turnover of molecules involved in immune sensing and pro-inflammatory signaling. Finding the right balance through selecting, stalling, and timing the cell response might be a powerful compensation for not being able to express effector molecules. While this is an exciting scenario that merits further investigation, experimental evidence for a direct link between virus induced membrane damage response and the (autophagic) control of the inflammatory response is currently lacking. The possibility that several additional TRIMs bind Gals offers the perspective that many more (TRIM) substrates could be recruited to membrane damage sites. The purpose of eliciting membrane damage for entering viruses could then be to remove as many cell effector molecules as possible and blunt the cells response to entry.

Taken together, there remains much to be discovered concerning our understanding how viruses penetrate cell membranes and how cells respond to this insult. Using the few existing examples presented in this review about the cell response to virus inflicted membrane damage, we invite the reader to reflect on the wider role of membrane damage for the virus.

## References

[1] Lombard J. (2014). Once upon a time the cell membranes: 175 years of cell boundary research. Biol. Direct.

[2] Thurston T.L.M.M., Wandel M.P., Von Muhlinen N., Foeglein Á., Randow F., Foeglein A., Randow F., Foeglein Á., Randow F. (2012). Galectin 8 targets damaged vesicles for autophagy to defend cells against bacterial invasion. Nature.

[3] Montespan C., Marvin S.A., Austin S., Burrage A.M., Roger B., Rayne F., Faure M., Campell E.M., Schneider C., Reimer R. (2017). Multi-layered control of Galectin-8 mediated autophagy during adenovirus cell entry through a conserved PPxY motif in the viral capsid. PLoS Pathog..

[4] Jia J., Abudu Y.P., Claude-Taupin A., Gu Y., Kumar S., Choi S.W., Peters R., Mudd M.H., Allers L., Salemi M. (2018). Galectins Control mTOR in Response to Endomembrane Damage. Mol. Cell.

[5] Vietri M., Radulovic M., Stenmark H. (2020). The many functions of ESCRTs. Nat. Rev. Mol. Cell Biol..

[6] Lozach P.Y., Huotari J., Helenius A. (2011). Late-penetrating viruses. Curr. Opin. Virol..

[7] Ketter E., Randall G. (2019). Virus Impact on Lipids and Membranes. Annu. Rev. Virol..

[8] Moyer C.L., Nemerow G.R. (2011). Viral weapons of membrane destruction: Variable modes of membrane penetration by non-enveloped viruses. Curr. Opin. Virol..

[9] Stewart P.L., Dermody T.S., Nemerow G.R. (2003). Structural basis of nonenveloped virus cell entry. Adv. Protein Chem..

[10] Prchla E., Plank C., Wagner E., Blaas D., Fuchs R. (1995). Virus-mediated release of endosomal content in vitro: Different behavior of adenovirus and rhinovirus serotype 2. J. Cell Biol..

[11] Farr G.A., Zhang L.G., Tattersall P. (2005). Parvoviral virions deploy a capsid-tethered lipolytic enzyme to breach the endosomal membrane during cell entry. Proc. Natl. Acad. Sci. USA.

[12] Greber U.F., Webster P., Weber J., Helenius A. (1996). The role of the adenovirus protease in virus entry into cells. EMBO J..

[13] Wiethoff C.M., Wodrich H., Gerace L., Nemerow G.R. (2005). Adenovirus Protein VI Mediates Membrane Disruption following Capsid Disassembly. J. Virol..

[14] Martinez R., Schellenberger P., Vasishtan D., Aknin C., Austin S., Dacheux D., Rayne F., Siebert A., Ruzsics Z., Gruenewald K. (2015). The amphipathic helix of adenovirus capsid protein VI contributes to penton release and postentry sorting. J. Virol..

[15] Weber J. (1976). Genetic analysis of adenovirus type 2 III. Temperature sensitivity of processing viral proteins. J. Virol..

[16] Cotten M., Weber J.M. (1995). The adenovirus protease is required for virus entry into host cells. Virology.

[17] Martín C.S. (2012). Latest insights on adenovirus structure and assembly. Viruses.

[18] Wodrich H., Henaff D., Jammart B., Segura-Morales C., Seelmeir S., Coux O., Ruzsics Z., Wiethoff C.M., Kremer E.J. (2010). A capsid-encoded PPxY-motif facilitates adenovirus entry. PLoS Pathog..

[19] Luisoni S., Suomalainen M., Boucke K., Tanner L.B., Wenk M.R., Guan X.L., Grzybek M., Coskun U., Greber U.F. (2015). Co-option of membrane wounding enables virus penetration into cells. Cell Host Microbe.

[20] Wiethoff C.M., Nemerow G.R. (2015). Adenovirus membrane penetration: Tickling the tail of a sleeping dragon. Virology.

[21] Hernando-Pérez M., Martín-González N., Pérez-Illana M., Suomalainen M., Condezo G.N., Ostapchuk P., Gallardo J., Menéndez M., Greber U.F., Hearing P. (2020). Dynamic competition for hexon binding between core protein VII and lytic protein VI promotes adenovirus maturation and entry. Proc. Natl. Acad. Sci. USA.

[22] Maier O., Galan D.L., Wodrich H., Wiethoff C.M. (2010). An N-terminal domain of adenovirus protein VI fragments membranes by inducing positive membrane curvature. Virology.

[23] Moyer C.L., Wiethoff C.M., Maier O., Smith J.G., Nemerow G.R. (2011). Functional Genetic and Biophysical Analyses of Membrane Disruption by Human Adenovirus. J. Virol..

[24] Pied N., Wodrich H. (2019). Imaging the adenovirus infection cycle. FEBS Lett..

[25] Dupzyk A., Tsai B. (2016). How polyomaviruses exploit the ERAD machinery to cause infection. Viruses.

[26] Magnuson B., Rainey E.K., Benjamin T., Baryshev M., Mkrtchian S., Tsai B. (2005). ERp29 triggers a conformational change in polyomavirus to stimulate membrane binding. Mol. Cell.

[27] Guerrero C.A., Bouyssounade D., Zárate S., Iša P., López T., Espinosa R., Romero P., Méndez E., López S., Arias C.F. (2002). Heat Shock Cognate Protein 70 Is Involved in Rotavirus Cell Entry. J. Virol..

[28] Walczak C.P., Ravindran M.S., Inoue T., Tsai B. (2014). A Cytosolic Chaperone Complexes with Dynamic Membrane J-Proteins and Mobilizes a Nonenveloped Virus out of the Endoplasmic Reticulum. PLoS Pathog..

[29] Inoue T., Tsai B. (2011). A large and intact viral particle penetrates the endoplasmic reticulum membrane to reach the cytosol. PLoS Pathog..

[30] Spriggs C.C., Badieyan S., Verhey K.J., Cianfrocco M.A., Tsai B. (2020). Golgi-associated BICD adaptors couple ER membrane penetration and disassembly of a viral cargo. J. Cell Biol..

[31] Suikkanen S., Antila M., Jaatinen A., Vihinen-Ranta M., Vuento M. (2003). Release of canine parvovirus from endocytic vesicles. Virology.

[32] Canaan S., Zádori Z., Ghomashchi F., Bollinger J., Sadilek M., Moreau M.E., Tijssen P., Gelb M.H. (2004). Interfacial Enzymology of Parvovirus Phospholipases A2. J. Biol. Chem..

[33] Dorsch S., Liebisch G., Kaufmann B., von Landenberg P., Hoffmann J.H., Drobnik W., Modrow S. (2002). The VP1 Unique Region of Parvovirus B19 and Its Constituent Phospholipase A2-Like Activity. J. Virol..

[34] Ebert D.H., Deussing J., Peters C., Dermody T.S. (2002). Cathepsin L and cathepsin B mediate reovirus disassembly in murine fibroblast cells. J. Biol. Chem..

[35] Chandran K., Farsetta D.L., Nibert M.L. (2002). Strategy for Nonenveloped Virus Entry: A Hydrophobic Conformer of the Reovirus Membrane Penetration Protein μ1 Mediates Membrane Disruption. J. Virol..

[36] Odegard A.L., Chandran K., Zhang X., Parker J.S.L., Baker T.S., Nibert M.L. (2004). Putative Autocleavage of Outer Capsid Protein μ1, Allowing Release of Myristoylated Peptide μ1N during Particle Uncoating, Is Critical for Cell Entry by Reovirus. J. Virol..

[37] Liemann S., Chandran K., Baker T.S., Nibert M.L., Harrison S.C. (2002). Structure of the reovirus membrane-penetration protein, μ1, in a complex with its protector protein, σ3. Cell.

[38] Zhang L., Agosto M.A., Ivanovic T., King D.S., Nibert M.L., Harrison S.C. (2009). Requirements for the Formation of Membrane Pores by the Reovirus Myristoylated μ1N Peptide. J. Virol..

[39] Agosto M.A., Ivanovic T., Nibert M.L. (2006). Mammalian reovirus, a nonfusogenic nonenveloped virus, forms size-selective pores in a model membrane. Proc. Natl. Acad. Sci. USA.

[40] Ivanovic T., Agosto M.A., Zhang L., Chandran K., Harrison S.C., Nibert M.L. (2008). Peptides released from reovirus outer capsid form membrane pores that recruit virus particles. EMBO J..

[41] Tuthill T.J., Groppelli E., Hogle J.M., Rowlands D.J. (2010). Picornaviruses. Current Topics in Microbiology and Immunology.

[42] Schober D., Kronenberger P., Prchla E., Blaas D., Fuchs R. (1998). Major and Minor Receptor Group Human Rhinoviruses Penetrate from Endosomes by Different Mechanisms. J. Virol..

[43] Panjwani A., Strauss M., Gold S., Wenham H., Jackson T., Chou J.J., Rowlands D.J., Stonehouse N.J., Hogle J.M., Tuthill T.J. (2014). Capsid Protein VP4 of Human Rhinovirus Induces Membrane Permeability by the Formation of a Size-Selective Multimeric Pore. PLoS Pathog..

[44] Staring J., Raaben M., Brummelkamp T.R. (2018). Viral escape from endosomes and host detection at a glance. J. Cell Sci..

[45] Staring J., Von Castelmur E., Blomen V.A., Van Den Hengel L.G., Brockmann M., Baggen J., Thibaut H.J., Nieuwenhuis J., Janssen H., Van Kuppeveld F.J.M.M. (2017). PLA2G16 represents a switch between entry and clearance of Picornaviridae. Nature.

[46] Hughes P.J., Stanway G. (2000). The 2A proteins of three diverse picornaviruses are related to each other and to the H-rev107 family of proteins involved in the control of cell proliferation. J. Gen. Virol..

[47] Knodler L.A., Vallance B.A., Celli J., Winfree S., Hansen B., Montero M., Steele-Mortimer O. (2010). Dissemination of invasive Salmonella via bacterial-induced extrusion of mucosal epithelia. Proc. Natl. Acad. Sci. USA.

[48] Mellouk N., Enninga J. (2016). Cytosolic access of intracellular bacterial pathogens: The Shigella paradigm. Front. Cell. Infect. Microbiol..

[49] Mellouk N., Weiner A., Aulner N., Schmitt C., Elbaum M., Shorte S.L., Danckaert A., Enninga J. (2014). Shigella subverts the host recycling compartment to rupture its vacuole. Cell Host Microbe.

[50] Weiner A., Mellouk N., Lopez-Montero N., Chang Y.-Y.Y., Souque C., Schmitt C., Enninga J. (2016). Macropinosomes are Key Players in Early Shigella Invasion and Vacuolar Escape in Epithelial Cells. PLoS Pathog..

[51] Birmingham C.L., Smith A.C., Bakowski M.A., Yoshimori T., Brumell J.H. (2006). Autophagy controls Salmonella infection in response to damage to the Salmonella-containing vacuole. J. Biol. Chem..

[52] Radtke A.L., Delbridge L.M., Balachandran S., Barber G.N., O’Riordan M.X.D. (2007). TBK1 protects vacuolar integrity during intracellular bacterial infection. PLoS Pathog..

[53] Boyle K.B., Thurston T.L.M., Randow F. (2016). TBK1 directs WIPI2 against Salmonella. Autophagy.

[54] Thurston T.L., Boyle K.B., Allen M., Ravenhill B.J., Karpiyevich M., Bloor S., Kaul A., Noad J., Foeglein A., Matthews S.A. (2016). Recruitment of TBK 1 to cytosol-invading Salmonella induces WIPI 2-dependent antibacterial autophagy. EMBO J..

[55] Santos J.C., Duchateau M., Fredlund J., Weiner A., Mallet A., Schmitt C., Matondo M., Hourdel V., Chamot-Rooke J., Enninga J. (2015). The COPII complex and lysosomal VAMP7 determine intracellular Salmonella localization and growth. Cell. Microbiol..

[56] Singh R., Jamieson A., Cresswell P. (2008). GILT is a critical host factor for Listeria monocytogenes infection. Nature.

[57] Radtke A.L., Anderson K.L., Davis M.J., DiMagno M.J., Swanson J.A., O’Riordan M.X. (2011). Listeria monocytogenes exploits cystic fibrosis transmembrane conductance regulator (CFTR) to escape the phagosome. Proc. Natl. Acad. Sci. USA.

[58] Gedde M.M., Higgins D.E., Tilney L.G., Portnoy D.A. (2000). Role of listeriolysin O in cell-to-cell spread of Listeria monocytogenes. Infect. Immun..

[59] Alberti-Segui C., Goeden K.R., Higgins D.E. (2007). Differential function of Listeria monocytogenes listeriolysin O and phospholipases C in vacuolar dissolution following cell-to-cell spread. Cell. Microbiol..

[60] Malet J.K., Cossart P., Ribet D. (2017). Alteration of epithelial cell lysosomal integrity induced by bacterial cholesterol-dependent cytolysins. Cell. Microbiol..

[61] Palframan S.L., Kwok T., Gabriel K. (2012). Vacuolating cytotoxin A (VacA), a key toxin for Helicobacter pylori pathogenesis. Front. Cell. Infect. Microbiol..

[62] Li F.Y., Weng I.C., Lin C.H., Kao M.C., Wu M.S., Chen H.Y., Liu F.T. (2018). Helicobacter pylori induces intracellular galectin-8 aggregation around damaged lysosomes within gastric epithelial cells in a host O-glycan-dependent manner. Glycobiology.

[63] van der Wel N., Hava D., Houben D., Fluitsma D., Van Zon M., Pierson J., Brenner M., Peters P.J.M. (2007). tuberculosis and M. leprae Translocate from the Phagolysosome to the Cytosol in Myeloid Cells. Cell.

[64] De Jonge M.I., Pehau-Arnaudet G., Fretz M.M., Romain F., Bottai D., Brodin P., Honoré N., Marchal G., Jiskoot W., England P. (2007). ESAT-6 from Mycobacterium tuberculosis dissociates from its putative chaperone CFP-10 under acidic conditions and exhibits membrane-lysing activity. J. Bacteriol..

[65] Akimana C., Al-Khodor S., Kwaik Y.A. (2010). Host factors required for modulation of phagosome biogenesis and proliferation of francisella tularensis within the cytosol. PLoS ONE.

[66] De Leon J., Jiang G., Ma Y., Rubin E., Fortune S., Sun J. (2012). Mycobacterium tuberculosis ESAT-6 exhibits a unique membrane-interacting activity that is not found in its ortholog from non-pathogenic Mycobacterium smegmatis. J. Biol. Chem..

[67] Peng X., Sun J. (2016). Mechanism of ESAT-6 membrane interaction and its roles in pathogenesis of Mycobacterium tuberculosis. Toxicon.

[68] Pilatz S., Breitbach K., Hein N., Fehlhaber B., Schulze J., Brenneke B., Eberl L., Steinmetz I. (2006). Identification of Burkholderia pseudomallei genes required for the intracellular life cycle and in vivo virulence. Infect. Immun..

[69] Whitworth T., Popov V.L., Yu X.J., Walker D.H., Bouyer D.H. (2005). Expression of the Rickettsia prowazekii pld or tlyC gene in Salmonella enterica serovar typhimurium mediates phagosomal escape. Infect. Immun..

[70] Renesto P., Dehoux P., Gouin E., Touqui L., Cossart P., Raoult D. (2003). Identification and Characterization of a Phospholipase D–Superfamily Gene in Rickettsiae. J. Infect. Dis..

[71] Clemens D.L., Lee B.Y., Horwitz M.A. (2004). Virulent and avirulent strains of Francisella tularensis prevent acidification and maturation of their phagosomes and escape into the cytoplasm in human macrophages. Infect. Immun..

[72] Clemens D.L., Ge P., Lee B.Y., Horwitz M.A., Zhou Z.H. (2015). Atomic structure of T6SS reveals interlaced array essential to function. Cell.

[73] Chong A., Wehrly T.D., Nair V., Fischer E.R., Barker J.R., Klose K.E., Celli J. (2008). The early phagosomal stage of Francisella tularensis determines optimal phagosomal escape and Francisella pathogenicity island protein expression. Infect. Immun..

[74] Berman J. (2006). Morphogenesis and cell cycle progression in Candida albicans. Curr. Opin. Microbiol..

[75] Westman J., Moran G., Mogavero S., Hube B., Grinsteina S. (2018). crossm Candida albicans Hyphal Expansion Causes Phagosomal. MBio.

[76] Vylkova S., Carman A.J., Danhof H.A., Collette J.R., Zhou H., Lorenz M.C. (2011). The fungal pathogen candida albicans autoinduces hyphal morphogenesis by raising extracellular pH. MBio.

[77] De Souza W., De Carvalho T.M.U., Barrias E.S. (2010). Review on Trypanosoma cruzi: Host cell interaction. Int. J. Cell Biol..

[78] Papadopoulos C., Kirchner P., Bug M., Grum D., Koerver L., Schulze N., Poehler R., Dressler A., Fengler S., Arhzaouy K. (2017). VCP/p97 cooperates with YOD 1, UBXD 1 and PLAA to drive clearance of ruptured lysosomes by autophagy. EMBO J..

[79] Freeman D., Cedillos R., Choyke S., Lukic Z., McGuire K., Marvin S., Burrage A.M., Sudholt S., Rana A., O’Connor C. (2013). Alpha-Synuclein Induces Lysosomal Rupture and Cathepsin Dependent Reactive Oxygen Species Following Endocytosis. PLoS ONE.

[80] Flavin W.P., Bousset L., Green Z.C., Chu Y., Skarpathiotis S., Chaney M.J., Kordower J.H., Melki R., Campbell E.M. (2017). Endocytic vesicle rupture is a conserved mechanism of cellular invasion by amyloid proteins. Acta Neuropathol..

[81] Villamil Giraldo A.M., Appelqvist H., Ederth T., Öllinger K. (2014). Lysosomotropic agents: Impact on lysosomal membrane permeabilization and cell death. Biochem. Soc. Trans..

[82] Aits S., Kricker J., Liu B., Ellegaard A.-M.M., Hämälistö S., Tvingsholm S., Corcelle-Termeau E., Høgh S., Farkas T., Jonassen A.H. (2015). Sensitive detection of lysosomal membrane permeabilization by lysosomal galectin puncta assay. Autophagy.

[83] Uchimoto T., Nohara H., Kamehara R., Iwamura M., Watanabe N., Kobayashi Y. (1999). Mechanism of apoptosis induced by a lysosomotropic agent, L-leucyl-L- leucine methyl ester. Apoptosis.

[84] Du Rietz H., Hedlund H., Wilhelmson S., Nordenfelt P., Wittrup A. (2020). Imaging small molecule-induced endosomal escape of siRNA. Nat. Commun..

[85] Wittrup A., Ai A., Liu X., Hamar P., Trifonova R., Charisse K., Manoharan M., Kirchhausen T., Lieberman J. (2015). Visualizing lipid-formulated siRNA release from endosomes and target gene knockdown. Nat. Biotechnol..

[86] Fraire J.C., Houthaeve G., Liu J., Raes L., Vermeulen L., Stremersch S., Brans T., García-Díaz Barriga G., De Keulenaer S., Van Nieuwerburgh F. (2020). Vapor nanobubble is the more reliable photothermal mechanism for inducing endosomal escape of siRNA without disturbing cell homeostasis. J. Control. Release.

[87] Chu Z., Miu K., Lung P., Zhang S., Zhao S., Chang H.C., Lin G., Li Q. (2015). Rapid endosomal escape of prickly nanodiamonds: Implications for gene delivery. Sci. Rep..

[88] Barondes S.H., Castronovo V., Cooper D.N.W., Cummings R.D., Drickamer K., Felzi T., Gitt M.A., Hirabayashi J., Hughes C., Kasai K.I. (1994). Galectins: A family of animal β-galactoside-binding lectins. Cell.

[89] Hirabayashi J., Kasai K.I. (1993). The family of metazoan metal-independent β-galactoside-binding lectins: Structure, function and molecular evolution. Glycobiology.

[90] Wang W.H., Lin C.Y., Chang M.R., Urbina A.N., Assavalapsakul W., Thitithanyanont A., Chen Y.H., Liu F.T., Wang S.F. (2019). The role of galectins in virus infection—A systemic literature review. J. Microbiol. Immunol. Infect..

[91] Baum L.G., Garner O.B., Schaefer K., Lee B. (2014). Microbe-host interactions are positively and negatively regulated by galectin-glycan interactions. Front. Immunol..

[92] Paz I., Sachse M., Dupont N., Mounier J., Cederfur C., Enninga J., Leffler H., Poirier F., Prevost M.C., Lafont F. (2010). Galectin-3, a marker for vacuole lysis by invasive pathogens. Cell. Microbiol..

[93] Maier O., Marvin S.A., Wodrich H., Campbell E.M., Wiethoff C.M. (2012). Spatiotemporal Dynamics of Adenovirus Membrane Rupture and Endosomal Escape. J. Virol..

[94] Martinez R., Burrage A.M., Wiethoff C.M., Wodrich H. (2013). High temporal resolution imaging reveals endosomal membrane penetration and escape of adenoviruses in real time. Methods Mol. Biol..

[95] Klionsky D.J., Codogno P. (2013). The mechanism and physiological function of macroautophagy. J. Innate Immun..

[96] Bento C.F., Renna M., Ghislat G., Puri C., Ashkenazi A., Vicinanza M., Menzies F.M., Rubinsztein D.C. (2016). Mammalian Autophagy: How Does It Work?. Annu. Rev. Biochem..

[97] Suzuki K., Noda T., Ohsumi Y. (2004). Interrelationships among Atg proteins during autophagy inSaccharomyces cerevisiae. Yeast.

[98] Klionsky D.J., Abdelmohsen K., Abe A., Abedin M.J., Abeliovich H., Acevedo Arozena A., Adachi H., Adams C.M., Adams P.D., Adeli K. (2016). Guidelines for the use and interpretation of assays for monitoring autophagy (3rd edition). Autophagy.

[99] Johansen T., Lamark T. (2011). Selective autophagy mediated by autophagic adapter proteins. Autophagy.

[100] Ravenhill B.J., Boyle K.B., von Muhlinen N., Ellison C.J., Masson G.R., Otten E.G., Foeglein A., Williams R., Randow F. (2019). The Cargo Receptor NDP52 Initiates Selective Autophagy by Recruiting the ULK Complex to Cytosol-Invading Bacteria. Mol. Cell.

[101] Vargas J.N.S., Wang C., Bunker E., Hao L., Maric D., Schiavo G., Randow F., Youle R.J. (2019). Spatiotemporal Control of ULK1 Activation by NDP52 and TBK1 during Selective Autophagy. Mol. Cell.

[102] Roberts R., Ktistakis N.T. (2013). Omegasomes: PI3P platforms that manufacture autophagosomes. Essays Biochem..

[103] Tanida I., Ueno T., Kominami E. (2004). LC3 conjugation system in mammalian autophagy. Int. J. Biochem. Cell Biol..

[104] Chauhan S., Kumar S., Jain A., Ponpuak M., Mudd M.H., Kimura T., Choi S.W., Peters R., Mandell M., Bruun J.A. (2016). TRIMs and Galectins Globally Cooperate and TRIM16 and Galectin-3 Co-direct Autophagy in Endomembrane Damage Homeostasis. Dev. Cell.

[105] Kimura T., Jia J., Kumar S., Choi S.W., Gu Y., Mudd M., Dupont N., Jiang S., Peters R., Farzam F. (2017). Dedicated SNARE s and specialized TRIM cargo receptors mediate secretory autophagy. EMBO J..

[106] Weng I.C., Chen H.L., Lo T.H., Lin W.H., Chen H.Y., Hsu D.K., Liu F.T. (2018). Cytosolic galectin-3 and -8 regulate antibacterial autophagy through differential recognition of host glycans on damaged phagosomes. Glycobiology.

[107] Feeley E.M., Pilla-Moffett D.M., Zwack E.E., Piro A.S., Finethy R., Kolb J.P., Martinez J., Brodsky I.E., Coers J. (2017). Galectin-3 directs antimicrobial guanylate binding proteins to vacuoles furnished with bacterial secretion systems. Proc. Natl. Acad. Sci. USA.

[108] Herhaus L., Dikic I. (2018). Regulation of Salmonella-host cell interactions via the ubiquitin system. Int. J. Med. Microbiol..

[109] Dupont N., Lacas-Gervais S., Bertout J., Paz I., Freche B., Van Nhieu G.T., van der Goot F.G., Sansonetti P.J., Lafont F. (2009). Shigella Phagocytic Vacuolar Membrane Remnants Participate in the Cellular Response to Pathogen Invasion and Are Regulated by Autophagy. Cell Host Microbe.

[110] Mostowy S., Sancho-Shimizu V., Hamon M.A., Simeone R., Brosch R., Johansen T., Cossart P. (2011). p62 and NDP52 proteins target intracytosolic Shigella and Listeria to different autophagy pathways. J. Biol. Chem..

[111] Luisoni S., Bauer M., Prasad V., Boucke K., Papadopoulos C., Meyer H., Hemmi S., Suomalainen M., Greber U. (2016). Endosomophagy clears disrupted early endosomes but not virus particles during virus entry into cells. Matters.

[112] Yoshida Y., Yasuda S., Fujita T., Hamasaki M., Murakami A., Kawawaki J., Iwai K., Saeki Y., Yoshimori T., Matsuda N. (2017). Ubiquitination of exposed glycoproteins by SCFFBXO27 directs damaged lysosomes for autophagy. Proc. Natl. Acad. Sci. USA.

[113] Fujita N., Morita E., Itoh T., Tanaka A., Nakaoka M., Osada Y., Umemoto T., Saitoh T., Nakatogawa H., Kobayashi S. (2013). Recruitment of the autophagic machinery to endosomes during infection is mediated by ubiquitin. J. Cell Biol..

[114] Tan J.M.M., Wong E.S.P., Kirkpatrick D.S., Pletnikova O., Ko H.S., Tay S.P., Ho M.W.L., Troncoso J., Gygi S.P., Lee M.K. (2008). Lysine 63-linked ubiquitination promotes the formation and autophagic clearance of protein inclusions associated with neurodegenerative diseases. Hum. Mol. Genet..

[115] Huett A., Heath R.J., Begun J., Sassi S.O., Baxt L.A., Vyas J.M., Goldberg M.B., Xavier R.J. (2012). The LRR and RING domain protein LRSAM1 is an E3 ligase crucial for ubiquitin-dependent autophagy of intracellular salmonella typhimurium. Cell Host Microbe.

[116] Noad J., Von Der Malsburg A., Pathe C., Michel M.A., Komander D., Randow F. (2017). LUBAC-synthesized linear ubiquitin chains restrict cytosol-invading bacteria by activating autophagy and NF-κB. Nat. Microbiol..

[117] Koerver L., Papadopoulos C., Liu B., Kravic B., Rota G., Brecht L., Veenendaal T., Polajnar M., Bluemke A., Ehrmann M. (2019). The ubiquitin-conjugating enzyme UBE 2 QL 1 coordinates lysophagy in response to endolysosomal damage. EMBO Rep..

[118] Seczynska M., Dikic I. (2017). Removing the waste bags: How p97 drives autophagy of lysosomes. EMBO J..

[119] Falcon B., Noad J., McMahon H., Randow F., Goedert M. (2018). Galectin-8-mediated selective autophagy protects against seeded tau aggregation. J. Biol. Chem..

[120] Richter B., Sliter D.A., Herhaus L., Stolz A., Wang C., Beli P., Zaffagnini G., Wild P., Martens S., Wagner S.A. (2016). Phosphorylation of OPTN by TBK1 enhances its binding to Ub chains and promotes selective autophagy of damaged mitochondria. Proc. Natl. Acad. Sci. USA.

[121] Thurston T.L.M., Ryzhakov G., Bloor S., von Muhlinen N., Randow F. (2009). The TBK1 adaptor and autophagy receptor NDP52 restricts the proliferation of ubiquitin-coated bacteria. Nat. Immunol..

[122] Pilli M., Arko-Mensah J., Ponpuak M., Roberts E., Master S., Mandell M.A., Dupont N., Ornatowski W., Jiang S., Bradfute S.B. (2012). TBK-1 Promotes Autophagy-Mediated Antimicrobial Defense by Controlling Autophagosome Maturation. Immunity.

[123] Kim J., Kundu M., Viollet B., Guan K.L. (2011). AMPK and mTOR regulate autophagy through direct phosphorylation of Ulk1. Nat. Cell Biol..

[124] Settembre C., Fraldi A., Medina D.L., Ballabio A., Children T. (2013). Signals from the lysosome. Nat. Rev. Mol. Cell Biol..

[125] Brady O.A., Martina J.A., Puertollano R. (2018). Emerging roles for TFEB in the immune response and inflammation. Autophagy.

[126] Nazio F., Carinci M., Valacca C., Bielli P., Strappazzon F., Antonioli M., Ciccosanti F., Rodolfo C., Campello S., Fimia G.M. (2016). Fine-tuning of ULK1 mRNA and protein levels is required for autophagy oscillation. J. Cell Biol..

[127] Jimenez A.J., Maiuri P., Lafaurie-Janvore J., Divoux S., Piel M., Perez F. (2014). ESCRT machinery is required for plasma membrane repair. Science.

[128] Scheffer L.L., Sreetama S.C., Sharma N., Medikayala S., Brown K.J., Defour A., Jaiswal J.K. (2014). Mechanism of Ca^2+^-triggered ESCRT assembly and regulation of cell membrane repair. Nat. Commun..

[129] Radulovic M., Schink K.O., Wenzel E.M., Nähse V., Bongiovanni A., Lafont F., Stenmark H. (2018). ESCRT-mediated lysosome repair precedes lysophagy and promotes cell survival. EMBO J..

[130] Hurley J.H. (2015). ESCRTs are everywhere. Embo J..

[131] Skowyra M.L., Schlesinger P.H., Naismith T.V., Hanson P.I. (2018). Triggered recruitment of ESCRT machinery promotes endolysosomal repair. Science.

[132] Repnik U., Distefano M.B., Speth M.T., Wui Ng M.Y., Progida C., Hoflack B., Gruenberg J., Griffiths G. (2017). L-leucyl-L-leucine methyl ester does not release cysteine cathepsins to the cytosol but inactivates them in transiently permeabilized lysosomes. J. Cell Sci..

[133] Jia J., Abudu Y.P., Claude-Taupin A., Gu Y., Kumar S., Choi S.W., Peters R., Mudd M.H., Allers L., Salemi M. (2019). Galectins Control MTOR and AMPK in Response to Lysosomal Damage to Induce Autophagy.

[134] Herbst S., Campbell P., Harvey J., Bernard E.M., Papayannopoulos V., Wood N.W., Morris H.R., Gutierrez M.G. (2020). LRRK2 activation controls the repair of damaged endomembranes in macrophages. EMBO J..

[135] Mittal E., Skowyra M.L., Uwase G., Tinaztepe E., Mehra A., Köster S., Hanson P.I., Philips J.A. (2018). Mycobacterium tuberculosis type VII secretion system effectors differentially impact the ESCRT endomembrane damage response. MBio.

[136] Kreibich S., Emmenlauer M., Fredlund J., Dehio C. (2015). Autophagy Proteins Promote Repair of Endosomal Membranes Damaged by the Salmonella Type Three Secretion System 1 Graphical Abstract Highlights d RNAi screen implicates autophagy in Salmonella-containing vacuole (SCV) maintenance d Autophagy promotes repair of TTSS-1-damaged SCV membranes and TTSS-2 expression d In parallel, autophagy eliminates cytosolic Salmonella d Autophagy could play a general role in maintaining endosomal membrane integrity. Cell Host Microbe.

[137] López-Jiménez A.T., Cardenal-Muñoz E., Leuba F., Gerstenmaier L., Barisch C., Hagedorn M., King J.S., Soldati T. (2018). The ESCRT and autophagy machineries cooperate to repair ESX-1-dependent damage at the Mycobacterium-containing vacuole but have opposite impact on containing the infection. PLOS Pathog..

[138] Hurley J.H., Emr S.D. (2006). The ESCRT complexes: Structure and mechanism of a membrane-trafficking network. Annu. Rev. Biophys. Biomol. Struct..

[139] Robinson M., Schor S., Barouch-Bentov R., Einav S. (2018). Viral journeys on the intracellular highways. Cell. Mol. Life Sci..

[140] Votteler J., Sundquist W.I. (2013). Virus budding and the ESCRT pathway. Cell Host Microbe.

[141] Ellison C.J., Kukulski W., Boyle K.B., Munro S., Randow F. (2020). Transbilayer Movement of Sphingomyelin Precedes Catastrophic Breakage of Enterobacteria-Containing Vacuoles. Curr. Biol..

[142] Gout E., Gutkowska M., Takayama S., Reed J.C., Chroboczek J. (2010). Co-chaperone BAG3 and adenovirus penton base protein partnership. J. Cell. Biochem..

[143] Zhao M., Antunes F., Eaton J.W., Brunk U.T. (2003). Lysosomal enzymes promote mitochondrial oxidant production, cytochrome c release and apoptosis. Eur. J. Biochem..

[144] Deus C.M., Yambire K.F., Oliveira P.J., Raimundo N. (2020). Mitochondria–Lysosome Crosstalk: From Physiology to Neurodegeneration. Trends Mol. Med..

[145] Christgen S., Place D.E., Kanneganti T.D. (2020). Toward targeting inflammasomes: Insights into their regulation and activation. Cell Res..

[146] Boya P., Kroemer G. (2008). Lysosomal membrane permeabilization in cell death. Oncogene.

[147] Aits S., Jäättelä M. (2013). Lysosomal cell death at a glance. J. Cell Sci..

[148] Deretic V., Saitoh T., Akira S. (2013). Autophagy in infection, inflammation and immunity. Nat Rev Immunol.

[149] Zhou R., Tardivel A., Thorens B., Choi I., Tschopp J. (2010). Thioredoxin-interacting protein links oxidative stress to inflammasome activation. Nat. Immunol..

[150] Zhou R., Yazdi A.S., Menu P., Tschopp J. (2011). A role for mitochondria in NLRP3 inflammasome activation. Nature.

[151] Kimura T., Jia J., Claude-Taupin A., Kumar S., Won Choi S., Gu Y., Mudd M., Dupont N., Jiang S., Peters R. (2017). Cellular and molecular mechanism for secretory autophagy. Autophagy.

[152] Brubaker S.W., Bonham K.S., Zanoni I., Kagan J.C. (2015). Innate Immune Pattern Recognition: A Cell Biological Perspective. Annu. Rev. Immunol..

[153] Kawai T., Akira S. (2011). Toll-like Receptors and Their Crosstalk with Other Innate Receptors in Infection and Immunity. Immunity.

[154] Gabandé-Rodríguez E., Pérez-Cañamás A., Soto-Huelin B., Mitroi D.N., Sánchez-Redondo S., Martínez-Sáez E., Venero C., Peinado H., Ledesma M.D. (2019). Lipid-induced lysosomal damage after demyelination corrupts microglia protective function in lysosomal storage disorders. EMBO J..

[155] Davis A.A., Leyns C.E.G., Holtzman D.M. (2018). Intercellular Spread of Protein Aggregates in Neurodegenerative Disease. Annu. Rev. Cell Dev. Biol..

[156] Schoonbroodt S., Ferreira V., Best-Belpomme M., Boelaert J.R., Legrand-Poels S., Korner M., Piette J. (2000). Crucial Role of the Amino-Terminal Tyrosine Residue 42 and the Carboxyl-Terminal PEST Domain of IκBα in NF-κB Activation by an Oxidative Stress. J. Immunol..

[157] Takada Y., Mukhopadhyay A., Kundu G.C., Mahabeleshwar G.H., Singh S., Aggarwal B.B. (2003). Hydrogen peroxide activates NF-κB through tyrosine phosphorylation of IκBα and serine phosphorylation of p65. Evidence for the involvement of IκBα kinase and Syk protein-tyrosine kinase. J. Biol. Chem..

[158] Anderson F.L., Coffey M.M., Berwin B.L., Havrda M.C. (2018). Inflammasomes: An Emerging Mechanism Translating Environmental Toxicant Exposure Into Neuroinflammation in Parkinson’s Disease. Toxicol. Sci..

[159] Siew J.J., Chen H.M., Chen H.Y., Chen H.L., Chen C.M., Soong B.W., Wu Y.R., Chang C.P., Chan Y.C., Lin C.H. (2019). Galectin-3 is required for the microglia-mediated brain inflammation in a model of Huntington’s disease. Nat. Commun..

[160] Streetly M.J., Maharaj L., Joel S., Schey S.A., Gribben J.G., Cotter F.E. (2010). GCS-100, a novel galectin-3 antagonist, modulates MCL-1, NOXA, and cell cycle to induce myeloma cell death. Blood.

[161] Kawasaki T., Kawai T. (2014). Toll-like receptor signaling pathways. Front. Immunol..

[162] O’Neill L.A.J., Golenbock D., Bowie A.G. (2013). The history of Toll-like receptors-redefining innate immunity. Nat. Rev. Immunol..

[163] Pichlmair A., Schulz O., Tan C.P., Näslund T.I., Liljeström P., Weber F., Reis E Sousa C. (2006). RIG-I-mediated antiviral responses to single-stranded RNA bearing 5′-phosphates. Science.

[164] Takahasi K., Yoneyama M., Nishihori T., Hirai R., Kumeta H., Narita R., Gale M., Inagaki F., Fujita T. (2008). Nonself RNA-Sensing Mechanism of RIG-I Helicase and Activation of Antiviral Immune Responses. Mol. Cell.

[165] Kato H., Takeuchi O., Sato S., Yoneyama M., Yamamoto M., Matsui K., Uematsu S., Jung A., Kawai T., Ishii K.J. (2006). Differential roles of MDA5 and RIG-I helicases in the recognition of RNA viruses. Nature.

[166] Zeng W., Sun L., Jiang X., Chen X., Hou F., Adhikari A., Xu M., Chen Z.J. (2010). Reconstitution of the RIG-I pathway reveals a signaling role of unanchored polyubiquitin chains in innate immunity. Cell.

[167] Loo Y.M., Gale M. (2011). Immune Signaling by RIG-I-like Receptors. Immunity.

[168] Pichlmair A., Reis e Sousa C. (2007). Innate Recognition of Viruses. Immunity.

[169] Goubau D., Schlee M., Deddouche S., Pruijssers A.J., Zillinger T., Goldeck M., Schuberth C., Van Der Veen A.G., Fujimura T., Rehwinkel J. (2014). Antiviral immunity via RIG-I-mediated recognition of RNA bearing 59-diphosphates. Nature.

[170] Gitlin L., Barchet W., Gilfillan S., Cella M., Beutler B., Flavell R.A., Diamond M.S., Colonna M. (2006). Essential role of mda-5 in type I IFN responses to polyriboinosinic: Polyribocytidylic acid and encephalomyocarditis picornavirus. Proc. Natl. Acad. Sci. USA.

[171] Feng Q., Hato S.V., Langereis M.A., Zoll J., Virgen-Slane R., Peisley A., Hur S., Semler B.L., van Rij R.P., van Kuppeveld F.J.M. (2012). MDA5 Detects the Double-Stranded RNA Replicative Form in Picornavirus-Infected Cells. Cell Rep..

[172] Wang J.P., Cerny A., Asher D.R., Kurt-Jones E.A., Bronson R.T., Finberg R.W. (2010). MDA5 and MAVS Mediate Type I Interferon Responses to Coxsackie B Virus. J. Virol..

[173] Slater L., Bartlett N.W., Haas J.J., Zhu J., Message S.D., Walton R.P., Sykes A., Dahdaleh S., Clarke D.L., Belvisi M.G. (2010). Co-ordinated role of TLR3, RIG-I and MDA5 in the innate response to rhinovirus in bronchial epithelium. PLoS Pathog..

[174] Ito M., Yanagi Y., Ichinohe T. (2012). Encephalomyocarditis Virus Viroporin 2B Activates NLRP3 Inflammasome. PLoS Pathog..

[175] Rajan J.V., Rodriguez D., Miao E.A., Aderem A. (2011). The NLRP3 Inflammasome Detects Encephalomyocarditis Virus and Vesicular Stomatitis Virus Infection. J. Virol..

[176] da Costa L.S., Outlioua A., Anginot A., Akarid K., Arnoult D. (2019). RNA viruses promote activation of the NLRP3 inflammasome through cytopathogenic effect-induced potassium efflux. Cell Death Dis..

[177] Smith J.S., Xu Z., Tian J., Palmer D.J., Ng P., Byrnes A.P. (2011). The role of endosomal escape and mitogen-activated protein kinases in adenoviral activation of the innate immune response. PLoS ONE.

[178] Nociari M., Ocheretina O., Murphy M., Falck-Pedersen E. (2009). Adenovirus Induction of IRF3 Occurs through a Binary Trigger Targeting Jun N-Terminal Kinase and TBK1 Kinase Cascades and Type I Interferon Autocrine Signaling. J. Virol..

[179] Stein S.C., Falck-Pedersen E. (2012). Sensing Adenovirus Infection: Activation of Interferon Regulatory Factor 3 in RAW 264.7 Cells. J. Virol..

[180] Eichholz K., Bru T., Tran T.T.P., Fernandes P., Welles H., Mennechet F.J.D., Manel N., Alves P., Perreau M., Kremer E.J. (2016). Immune-Complexed Adenovirus Induce AIM2-Mediated Pyroptosis in Human Dendritic Cells. PLoS Pathog..

[181] Barlan A.U., Griffin T.M., Mcguire K.A., Wiethoff C.M. (2011). Adenovirus Membrane Penetration Activates the NLRP3 Inflammasome. J. Virol..

[182] McGuire K.A., Barlan A.U., Griffin T.M., Wiethoff C.M. (2011). Adenovirus Type 5 Rupture of Lysosomes Leads to Cathepsin B-Dependent Mitochondrial Stress and Production of Reactive Oxygen Species. J. Virol..

[183] Tibbles L.A., Spurrell J.C.L., Bowen G.P., Liu Q., Lam M., Zaiss A.K., Robbins S.M., Hollenberg M.D., Wickham T.J., Muruve D.A. (2002). Activation of p38 and ERK Signaling during Adenovirus Vector Cell Entry Lead to Expression of the C-X-C Chemokine IP-10. J. Virol..

[184] Anghelina D., Lam E., Falck-Pedersen E. (2016). Diminished Innate Antiviral Response to Adenovirus Vectors in cGAS/STING-Deficient Mice Minimally Impacts Adaptive Immunity. J. Virol..

[185] Maler M.D., Nielsen P.J., Stichling N., Cohen I., Ruzsics Z., Wood C., Engelhard P., Suomalainen M., Gyory I., Huber M. (2017). Key role of the scavenger receptor MARCO in mediating adenovirus infection and subsequent innate responses of macrophages. MBio.

[186] Watkinson R.E., McEwan W.A., Tam J.C.H., Vaysburd M., James L.C. (2015). TRIM21 Promotes cGAS and RIG-I Sensing of Viral Genomes during Infection by Antibody-Opsonized Virus. PLoS Pathog..

[187] Hare D., Mossman K.L. (2013). Novel paradigms of innate immune sensing of viral infections. Cytokine.

[188] Collins S.E., Mossman K.L. (2014). Danger, diversity and priming in innate antiviral immunity. Cytokine Growth Factor Rev..

[189] Deretic V. (2012). Autophagy as an innate immunity paradigm: Expanding the scope and repertoire of pattern recognition receptors. Curr. Opin. Immunol..

[190] Saitoh T., Fujita N., Jang M.H., Uematsu S., Yang B.G., Satoh T., Omori H., Noda T., Yamamoto N., Komatsu M. (2008). Loss of the autophagy protein Atg16L1 enhances endotoxin-induced IL-1β production. Nature.

[191] Santeford A., Wiley L.A., Park S., Bamba S., Nakamura R., Gdoura A., Ferguson T.A., Rao P.K., Guan J.L., Saitoh T. (2016). Impaired autophagy in macrophages promotes inflammatory eye disease. Autophagy.

[192] Pu Q., Gan C., Li R., Li Y., Tan S., Li X., Wei Y., Lan L., Deng X., Liang H. (2017). Atg7 Deficiency Intensifies Inflammasome Activation and Pyroptosis in Pseudomonas Sepsis. J. Immunol..

[193] Bechelli J., Vergara L., Smalley C., Buzhdygan T.P., Bender S., Zhang W., Liu Y., Popov V.L., Wang J., Garg N. (2019). Atg5 supports Rickettsia australis infection in macrophages in vitro and in vivo. Infect. Immun..

[194] Nakahira K., Haspel J.A., Rathinam V.A.K., Lee S.J., Dolinay T., Lam H.C., Englert J.A., Rabinovitch M., Cernadas M., Kim H.P. (2011). Autophagy proteins regulate innate immune responses by inhibiting the release of mitochondrial DNA mediated by the NALP3 inflammasome. Nat. Immunol..

[195] Zhong Z., Umemura A., Sanchez-Lopez E., Liang S., Shalapour S., Wong J., He F., Boassa D., Perkins G., Ali S.R. (2016). NF-κB Restricts Inflammasome Activation via Elimination of Damaged Mitochondria. Cell.

[196] Sumpter R., Sirasanagandla S., Fernández Á.F., Wei Y., Dong X., Franco L., Zou Z., Marchal C., Lee M.Y., Clapp D.W. (2016). Fanconi Anemia Proteins Function in Mitophagy and Immunity. Cell.

[197] Nguyen T.N., Padman B.S., Lazarou M. (2016). Deciphering the Molecular Signals of PINK1/Parkin Mitophagy. Trends Cell Biol..

[198] Chen G., Kroemer G., Kepp O. (2020). Mitophagy: An Emerging Role in Aging and Age-Associated Diseases. Front. Cell Dev. Biol..

[199] Harris J., Hartman M., Roche C., Zeng S.G., O’Shea A., Sharp F.A., Lambe E.M., Creagh E.M., Golenbock D.T., Tschopp J. (2011). Autophagy controls IL-1β secretion by targeting Pro-IL-1β for degradation. J. Biol. Chem..

[200] Shi C.-S.S., Shenderov K., Huang N.-N.N., Kabat J., Abu-Asab M., Fitzgerald K.A., Sher A., Kehrl J.H. (2012). Activation of autophagy by inflammatory signals limits IL-1β production by targeting ubiquitinated inflammasomes for destruction. Nat. Immunol..

[201] Liu T., Tang Q., Liu K., Xie W., Liu X., Wang H., Wang R.F., Cui J. (2016). TRIM11 Suppresses AIM2 Inflammasome by Degrading AIM2 via p62-Dependent Selective Autophagy. Cell Rep..

[202] Kimura T., Jain A., Choi S.W., Mandell M.A., Schroder K., Johansen T., Deretic V. (2015). TRIM-mediated precision autophagy targets cytoplasmic regulators of innate immunity. J. Cell Biol..

[203] Van Gent M., Sparrer K.M.J., Gack M.U. (2018). TRIM proteins and their roles in antiviral host defenses. Annu. Rev. Virol..

[204] Jounai N., Kobiyama K., Shiina M., Ogata K., Ishii K.J., Takeshita F. (2011). NLRP4 Negatively Regulates Autophagic Processes through an Association with Beclin1. J. Immunol..

[205] Dupont N., Jiang S., Pilli M., Ornatowski W., Bhattacharya D., Deretic V. (2011). Autophagy-based unconventional secretory pathway for extracellular delivery of IL-1β. EMBO J..

[206] Zhang M., Kenny S.J., Ge L., Xu K., Schekman R. (2015). Translocation of interleukin-1β into a vesicle intermediate in autophagy-mediated secretion. Elife.

[207] Wang L.J., Huang H.Y., Huang M.P., Liou W., Chang Y.T., Wu C.C., Ojcius D.M., Chang Y.S. (2014). The microtubule-associated protein EB1 links AIM2 inflammasomes with autophagy-dependent secretion. J. Biol. Chem..

[208] Deretic V. (2009). Multiple regulatory and effector roles of autophagy in immunity. Curr. Opin. Immunol..

[209] Xu Y., Liu X.-D., Gong X., Eissa N.T. (2008). Signaling pathway of autophagy associated with innate immunity. Autophagy.

[210] Delgado M.A., Elmaoued R.A., Davis A.S., Kyei G., Deretic V. (2008). Toll-like receptors control autophagy. EMBO J..

[211] Shi C.-S., Kehrl J.H. (2008). MyD88 and Trif target Beclin 1 to trigger autophagy in macrophages. J. Biol. Chem..

[212] Jounai N., Takeshita F., Kobiyama K., Sawano A., Miyawaki A., Xin K.Q., Ishii K.J., Kawai T., Akira S., Suzuki K. (2007). The Atg5-Atg12 conjugate associates with innate antiviral immune responses. Proc. Natl. Acad. Sci. USA.

[213] Jin S., Tian S., Chen Y., Zhang C., Xie W., Xia X., Cui J., Wang R. (2016). USP 19 modulates autophagy and antiviral immune responses by deubiquitinating Beclin-1. EMBO J..

[214] Jin S., Tian S., Luo M., Xie W., Liu T., Duan T., Wu Y., Cui J. (2017). Tetherin Suppresses Type I Interferon Signaling by Targeting MAVS for NDP52-Mediated Selective Autophagic Degradation in Human Cells. Mol. Cell.

[215] Du Y., Duan T., Feng Y., Liu Q., Lin M., Cui J., Wang R. (2018). LRRC25 inhibits type I IFN signaling by targeting ISG15-associated RIG-I for autophagic degradation. EMBO J..

[216] Saitoh T., Fujita N., Hayashi T., Takahara K., Satoh T., Lee H., Matsunaga K., Kageyama S., Omori H., Noda T. (2009). Atg9a controls dsDNA-driven dynamic translocation of STING and the innate immune response. Proc. Natl. Acad. Sci. USA.

[217] Liang Q., Seo G.J., Choi Y.J., Kwak M.J., Ge J., Rodgers M.A., Shi M., Leslie B.J., Hopfner K.P., Ha T. (2014). Crosstalk between the cGAS DNA sensor and beclin-1 autophagy protein shapes innate antimicrobial immune responses. Cell Host Microbe.

[218] Chen M., Meng Q., Qin Y., Liang P., Tan P., He L., Zhou Y., Chen Y., Huang J., Wang R.F. (2016). TRIM14 Inhibits cGAS Degradation Mediated by Selective Autophagy Receptor p62 to Promote Innate Immune Responses. Mol. Cell.

[219] Tsuchida T., Zou J., Saitoh T., Kumar H., Abe T., Matsuura Y., Kawai T., Akira S. (2010). The ubiquitin ligase TRIM56 regulates innate immune responses to intracellular double-stranded DNA. Immunity.

[220] Prabakaran T., Bodda C., Krapp C., Zhang B., Christensen M.H., Sun C., Reinert L., Cai Y., Jensen S.B., Skouboe M.K. (2018). Attenuation of c GAS-STING signaling is mediated by a p62/ SQSTM 1-dependent autophagy pathway activated by TBK1. EMBO J..

[221] Niida M., Tanaka M., Kamitani T. (2010). Downregulation of active IKKβ by Ro52-mediated autophagy. Mol. Immunol..

[222] Zheng Q., Hou J., Zhou Y., Yang Y., Xie B., Cao X. (2015). Siglec1 suppresses antiviral innate immune response by inducing TBK1 degradation via the ubiquitin ligase TRIM27. Cell Res..

[223] Sparrer K.M.J., Gableske S., Zurenski M.A., Parker Z.M., Full F., Baumgart G.J., Kato J., Pacheco-Rodriguez G., Liang C., Pornillos O. (2017). TRIM23 mediates virus-induced autophagy via activation of TBK1. Nat. Microbiol..

[224] Mandell M.A., Jain A., Arko-Mensah J., Chauhan S., Kimura T., Dinkins C., Silvestri G., Münch J., Kirchhoff F., Simonsen A. (2014). TRIM proteins regulate autophagy and can target autophagic substrates by direct recognition. Dev. Cell.

[225] Labzin L.I., Bottermann M., Rodriguez-Silvestre P., Foss S., Andersen J.T., Vaysburd M., Clift D., James L.C. (2019). Antibody and DNA sensing pathways converge to activate the inflammasome during primary human macrophage infection. EMBO J..

[226] Klein K.A., Jackson W.T. (2011). Picornavirus subversion of the autophagy pathway. Viruses.

